# Immune checkpoint inhibitors in Cancer patients with rheumatologic preexisting autoimmune diseases: a systematic review and meta-analysis

**DOI:** 10.1186/s12885-024-12256-z

**Published:** 2024-04-17

**Authors:** Xin Liu, Su Li, Liyuan Ke, Hongxia Cui

**Affiliations:** 1 Department of Colorectal Surgery, Cancer Hospital of China Medical University, Liaoning Cancer Hospital and Institute, Shenyang, China; 2 Department of Pharmacy, Cancer Hospital of China Medical University, Liaoning Cancer Hospital and Institute, Shenyang, China

**Keywords:** Rheumatologic preexisting autoimmune diseases, Immune checkpoint inhibitors, Immune-related adverse events, Meta-analysis

## Abstract

**Background:**

Patients with rheumatologic preexisting autoimmune disease (PAD) have not been enrolled in clinical trials of immune checkpoint inhibitors (ICIs). Therefore, the risks and benefits of ICI therapy in such patients are unclear. Herein, we investigated the safety and efficacy of ICIs in rheumatologic PAD patients through a meta-analysis.

**Methods:**

The PubMed, Cochrane Library, Embase and Web of Science databases were searched for additional studies. We analyzed the following data through Stata software: incidence of total irAEs (TirAEs), rate of flares, incidence of new on-set irAEs, rate of discontinuation, objective response rate (ORR) and disease control rate (DCR).

**Results:**

We identified 23 articles including 643 patients with rheumatologic PAD. The pooled incidences of TirAEs, flares and new-onset irAEs were 64% (95% CI 55%-72%), 41% (95% CI 31%-50%), and 33% (95% CI 28%-38%), respectively. In terms of severity, the incidences were 7% (95% CI 2%-14%) for Grade 3–4 flares and 12% (95% CI 9%-15%) for Grade 3–4 new-onset irAEs. Patients with RA had a greater risk of flares than patients with other rheumatologic PADs did (RR = 1.35, 95% CI 1.03–1.77). The ORR and DCR were 30% and 44%, respectively. Baseline anti-rheumatic treatment was not significantly associated with the frequency of flares (RR = 1.05, 95% CI 0.63–1.77) or the ORR (RR = 0.45, 95% CI 0.12–1.69).

**Conclusions:**

Patients with rheumatologic PAD, particularly those with RA, are susceptible to relapse of their rheumatologic disease following ICI therapy. ICIs are also effective for treating rheumatologic PAD patients.

**Prospective register of systematic reviews (PROSPERO):**

number CRD 42,023,439,702.

**Supplementary Information:**

The online version contains supplementary material available at 10.1186/s12885-024-12256-z.

## Introduction

In recent years, immune checkpoint inhibitors (ICIs) targeting checkpoints such as cytotoxic T-lymphocyte-associated-4 (CTLA-4), programmed cell death 1 (PD-1), and programmed death-ligand 1 (PD-L1) have been found to be essential components in the treatment of a wide range of cancers [1–3]. The binding of PD-1 to its ligand PD-L1 and the binding of CTLA-4 to its ligand CD80/CD86 downregulate T-cell activation, leading to immune escape of tumor cells [4]. ICIs can boost the activation of T cells by blocking the engagement of the above receptors and ligands [5]. Although the survival benefit of ICIs is well recognized, they also result in immune-related adverse events (irAEs) [6], especially in patients with preexisting autoimmune diseases (PADs).

Rheumatologic PAD is a common PAD associated with cancer. Compared with the general population, patients with rheumatologic PADs, such as rheumatoid arthritis (RA) [7], systemic sclerosis [8] and Sjogren’s syndrome [9], have an increased risk of specific cancers. It is worth noting that some patients suffer from both rheumatologic PAD and cancer. A study by Khan et al. included 210,509 lung cancer patients, of which 9.4% also had rheumatologic PAD [10]. Studies have reported that the PD-1 pathway and CTLA-4 pathway may play potential roles in the occurrence and development of rheumatologic PAD [11–13]. These findings suggested that ICIs may theoretically increase the risk of rheumatologic PAD flares. For this reason, patients with rheumatologic PAD have largely been excluded from clinical trials. At present, available evidence regarding the exact incidence of disease flares, new-onset irAEs or cancer treatment outcomes in this population remains scarce. Considering the increasing dependence on ICIs for the treatment of tumors and the high proportion of cancer patients with rheumatologic PAD, it is especially essential to explore the influence of rheumatologic PAD on ICI treatment outcomes.

Hitherto, there has been increasing interest in the eligibility of receiving ICIs in patients with PAD, and several meta-analyses have assessed the safety and effectiveness of ICIs in patients with cancer and PAD [14–16]. However, these studies focused on a wide variety of autoimmune diseases, and none of them separately reported the incidence of rheumatic disease flares, new-onset irAEs or ICI efficacy in cancer patients with preexisting autoimmune rheumatic diseases. Although multiple studies have reported the efficacy and safety of using ICIs in these patients, a definite conclusion has not been reached according to the results of each single study [17–39]. Based on the above background, our study aimed (i) to summarize the incidence of rheumatologic PAD flares, the incidence of new-onset irAEs, and the rate of discontinuation; (ii) to investigate the objective response rate (ORR) or disease control rate (DCR); and (iii) to explore the impact of baseline anti-rheumatic treatment on the efficacy and safety of ICI treatment in patients with rheumatologic PAD and cancer.

## Methods

This systematic review was reported in accordance with the Preferred Reporting Items for Systematic Reviews and Meta-Analyses (PRISMA) guidelines. The study has been registered in the International Prospective Register of Systematic Reviews (PROSPERO), number CRD 42,023,439,702.

### Search strategy

We searched the PubMed, Embase, Cochrane Library, and Web of Science databases from database inception to June 1, 2023. Two researchers (CHX and LX) independently searched available studies by a combination of four themes, namely, neoplasm, immune checkpoint inhibitors, autoimmune rheumatic diseases, and preexisting diseases. The detailed search strategy is provided in Supplementary Table 1. We also manually searched the references of each relevant article to identify additional relevant studies.

### Selection criteria

The inclusion criteria were as follows: (1) P: patients were limited to those with cancer and rheumatologic PAD; (2) I: patients who received ICIs; (3) O: studies reporting the safety or efficacy of ICIs in patients with rheumatologic PAD; and (4) S: prospective or retrospective studies. The exclusion criteria for patients were as follows: (1) studies in which relevant data could not be obtained; (2) review articles, case reports, conference abstracts, comments, meta-analyses, or letters; and (3) duplicate studies. If multiple publications reporting the same population were found, the article with the most updated or comprehensive irAE information was selected.

### Data extraction and quality assessment

Two researchers (CHX and LX) extracted the following data from the included studies: (1) Basic information: author, publication year, region, sample size, type of cancer and type of ICI, and rheumatologic disease subtype; (2) Outcome: number of irAEs, discontinuation and response, including complete response (CR), partial response (PR) and stable disease (SD) (ORR = CR + PR, DCR = CR + PR + SD). Considering the classification, irAEs included three categories: flares, new-onset irAEs and total irAEs (TirAEs). The flares of PAD were defined as worsening or exacerbation of PAD after ICIs therapy, the new-onset irAEs was newly developed irAEs that did not have a clear causal relationship with PAD, and the TirAEs was flares, new-onset irAEs or both. All irAEs including flares, new-onset irAEs and TirAEs were graded according to the Common Toxicity Criteria for Adverse Events (CTCAE) in all original literature. We assessed the quality of the studies through two tools based on the study design. First, the Newcastle–Ottawa Scale (NOS) was used for cohort and case‒control studies [40]. While the scores of the included studies were 0–3, 4–6, and 7–9, these studies were considered to be of low, medium, and high quality, respectively. Second, the Joanna Briggs Institute (JBI) checklist was applied for case series [41]. The quality evaluation of the case series consisted of 10 items. Each item requires a response of yes, no, unclear, or not applicable. Any disagreements were resolved by discussion.

### Statistical analysis

We combined the pooled incidence rate with the 95% CI for flares, new-onset irAEs, and TirAEs and the pooled response rate with the 95% CI among ICI-treated patients with rheumatic diseases. The heterogeneity was tested using the I^2^ statistic. A meta-analysis was carried out using a random-effects model if heterogeneity existed. We further identified the sources of heterogeneity by meta-regression and subgroup analyses based on region, type of cancer, type of ICI and rheumatologic disease subtype. In addition, publication bias was assessed using Begg’s and Egger’s tests. All the data were analyzed with Stata 14.0 (Stata Corp.). A *P* value < 0.05 indicated statistical significance.

## Results

### Selection of studies and characteristics of the included studies

**Fig. 1:**
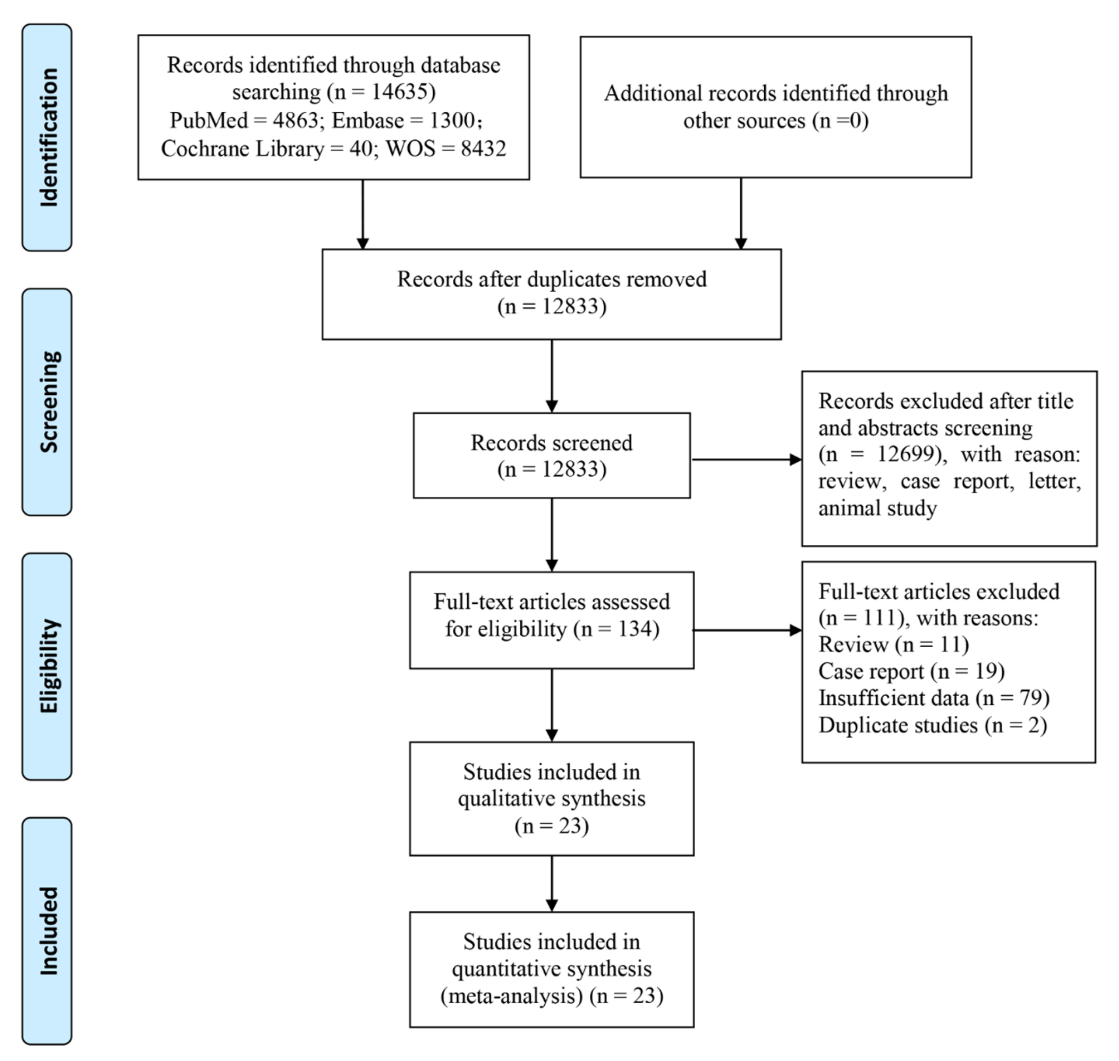
Diagram of the study selection process

According to the search strategy, we identified 14,635 records. After deleting duplicate records and ineligible articles such as case reports, reviews, letters, and animal studies, the full texts of the remaining 134 studies were screened. Of these, 111 studies were inappropriate because they did not meet the inclusion criteria. Finally, 23 articles [17–39] comprising a total of 653 patients were included in the analysis (Fig. 1). Concerning regions, five of the studies were from multiple nations, nine from North America, nine from Europe, and the remaining two from Australia. Of these, 13 studies investigated melanoma, two studies focused on urological cancer, one study evaluated non-small cell lung cancer (NSCLC), and the remaining studies were mixed. The types of ICIs used included anti-PD-1/PD-L1, anti-CTLA-4 and mixed ICIs. In terms of the types of rheumatologic diseases, most studies analyzed various rheumatologic diseases; three studies included RA patients only, and one study focused on systemic sclerosis. The median follow-up time ranged from 4.7 to 27 months. The main features of the 23 included studies are described in Table 1, and additional information is summarized in Supplementary Table 2. The NOS scores of the four cohort studies ranged from 6 to 9, indicating that the cohort studies were medium- to high-quality studies (Supplementary Table 3). Seven out of 19 case series met all the criteria of the JBI, suggesting that the above seven studies were of good quality (Supplementary Table 4).

**Table 1:** The main characteristics of the studies included in the meta-analysis

Author and year	Patients (n)	TirAEs (%)	Flare, n(%)	G1-2, n(%)	G3-4, n(%)	New onset irAEs n(%)	G1-2, n(%)	G3-4, n(%)	Discont-inuation, n(%)	ORR (%)	DCR (%)
Bhatlapenumarthi-2020 [17]	24	NA	5(20.8%)	NA	NA	NA	NA	NA	NA	NA	NA
Brown-2021 [18]	11	NA	7(63.6%)	5(45.5%)	2(18.2%)	5(45.5%)	NA	NA	NA	45.5%	54.5%
Cortellini-2019 [19]	10	5(50%)	1(10.0%)	1(10.0%)	0	4(40.0%)	4(40.0%)	0	NA	NA	NA
Danlos-2018 [20]	7	NA	1(14.3%)	NA	NA	NA	NA	NA	NA	NA	NA
Efuni-2021 [21]	22	16(72.7%)	12(54.5%)	NA	NA	7(31.8%)	5(22.7%)	2(9.1%)	9(40.9%)	NA	NA
Gutzmer-2017 [22]	9	7(77.8%)	5(55.6%)	3(33.3%)	2(22.2%)	2(22.2%)	2(22.2%)	0	0	44.4%	44.4%
Hoa-2021 [23]	19	12(63.2%)	8(42.1%)	8(42.1%)	0	7(36.8%)	4(21.0%)	3(15.8%)	NA	NA	42.1%
Johnson-2016 [24]	10	7(70.0%)	5(50.0%)	NA	NA	3(30.0%)	0	3(30.0%)	NA	30.0%	NA
Kähler-2017 [25]	14	7(50.0%)	6(42.9%)	NA	NA	4(28.6%)	2(14.3%)	2(14.3%)	1(7.1%)	0	0
Kaur-2019 [26]	5	NA	1(20.0%)	NA	NA	NA	NA	NA	0	NA	NA
Lee-2016 [27]	8	8(100.0%)	6(75.0%)	4(50.0%)	2(25.0%)	NA	NA	4(50.0%)	5(62.5%)	50.0%	87.5%
Leonardi-2018 [28]	25	12(48.0%)	10(40.0%)	7(28.0%)	2(8.0%)	6(24.0%)	4(16.0%)	2(8.0%)	6(24.0%)	NA	NA
Loriot-2020 [29]	7	NA	1(14.3%)	0	1(14.3%)	NA	NA	NA	NA	NA	NA
Lusa-2022 [30]	45	27(60.0%)	13(28.9%)	13(28.9%)	0	20(44.4%)	NA	8(17.8%)	16(35.6%)	13.3%	17.8%
Machado-2023 [31]	58	NA	15(25.9%)	NA	NA	14(24.1%)	NA	NA	NA	NA	NA
Martinez Chanza-2020 [32]	35	23(65.7%)	18(51.4%)	NA	NA	13(37.1%)	NA	NA	NA	NA	NA
Menzies-2016 [33]	27	NA	14(51.9%)	NA	NA	NA	NA	NA	NA	NA	NA
Mitchell-2018 [34]	12	10(83.3%)	10(83.3%)	8(66.7%)	2(16.7%)	3(25.0%)	1(8.3%)	2(16.7%)	3(25.0%)	50.0%	66.7%
Mooradian-2019 [35]	6	NA	6(100%)	NA	NA	2(33.3%)	NA	NA	2(33.3%)	33.3%	66.7%
Panhaleux-2020 [36]	17	NA	4(23.5%)	0	4(23.5%)	10(58.8%)	9(52.9%)	1(5.9%)	NA	41.2%	58.8%
Richter-2017 [37]	16	6(37.5%)	1(6.25%)	1(6.2%)	0	5(31.3%)	1(6.25%)	4(25.0%)	6(37.5%)	NA	NA
Tison-2019 [38]	39	23(58.9%)	19(48.7%)	13(40.6%)	3(9.4%)	NA	NA	NA	6(15.3%)	NA	NA
Van der Kooij-2021 [39]	227	NA	NA	NA	NA	NA	NA	29(12.8%)	NA	NA	NA

Abbreviations: TirAEs, flare, new onset irAEs or both; G1-2, Grade 1 or 2; G3–4, Grade 3 or 4; ORR, objective response rate; DCR, disease control rate; NA, not available

### The safety of ICIs in treating rheumatologic PAD

#### The incidence of TirAEs

**Fig. 2:**
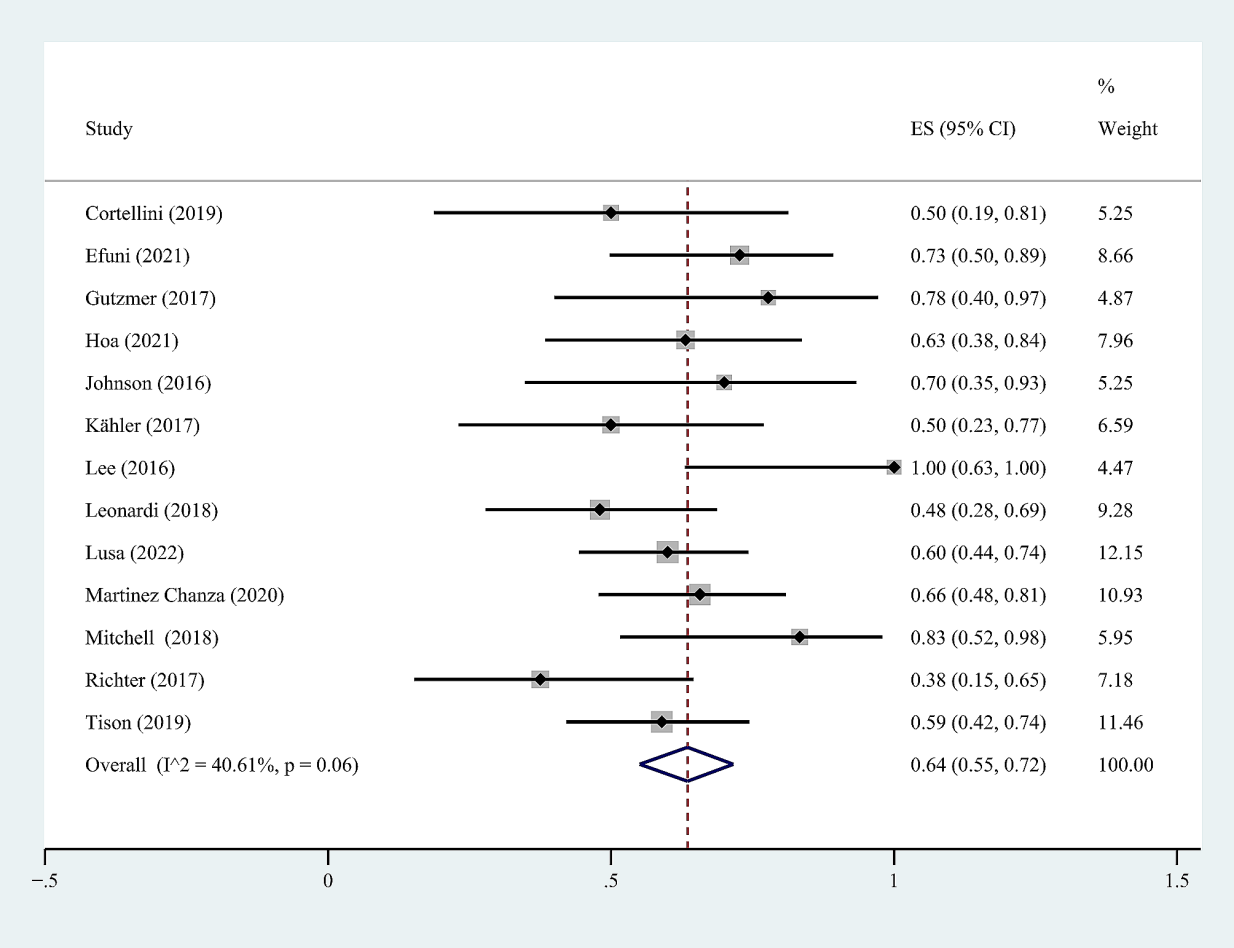
The pooled incidence rate of any-grade TirAEs

As illustrated in Fig. 2, a total of 13 studies [19, 21–25, 27, 28, 30, 32, 34, 37, 38] reported that 163 out of 264 patients with rheumatologic PAD experienced TirAEs. The incidence of TirAEs ranged from 37.5 to 100%, with a crude incidence of 61%. According to a random effects model, the summary results showed that the pooled incidence of TirAEs was 64% (95% CI 55%-72%, I^2^ = 40.61%). Sometimes, the same patient may experience both flares and new-onset irAEs with different grades of adverse reactions, making it impossible to grade the TirAEs. Therefore, we did not analyze the incidence of Grade 1–2 or Grade 3–4 TirAEs.

To further investigate the impact of region and type of ICI on the results, subgroup analyses were carried out. According to our subgroup analyses based on the type of ICI, there was no significant difference in the incidence of TirAEs between the subgroups. However, in the subgroup analysis by region, we found that the incidence of TirAEs in the Australian population was significantly greater than that in the other regions (*P* = 0.014); the incidence of TirAEs was 57% (95% CI 46%-68%) in North America, 59% (95% CI 46%-70%) in Europe, 67% (95% CI 52%-81%) in the multinational group, and 93% (95% CI 75%-100%) in Australia. (Supplementary Fig. 1).

#### Flares of rheumatologic diseases

**Fig. 3:**
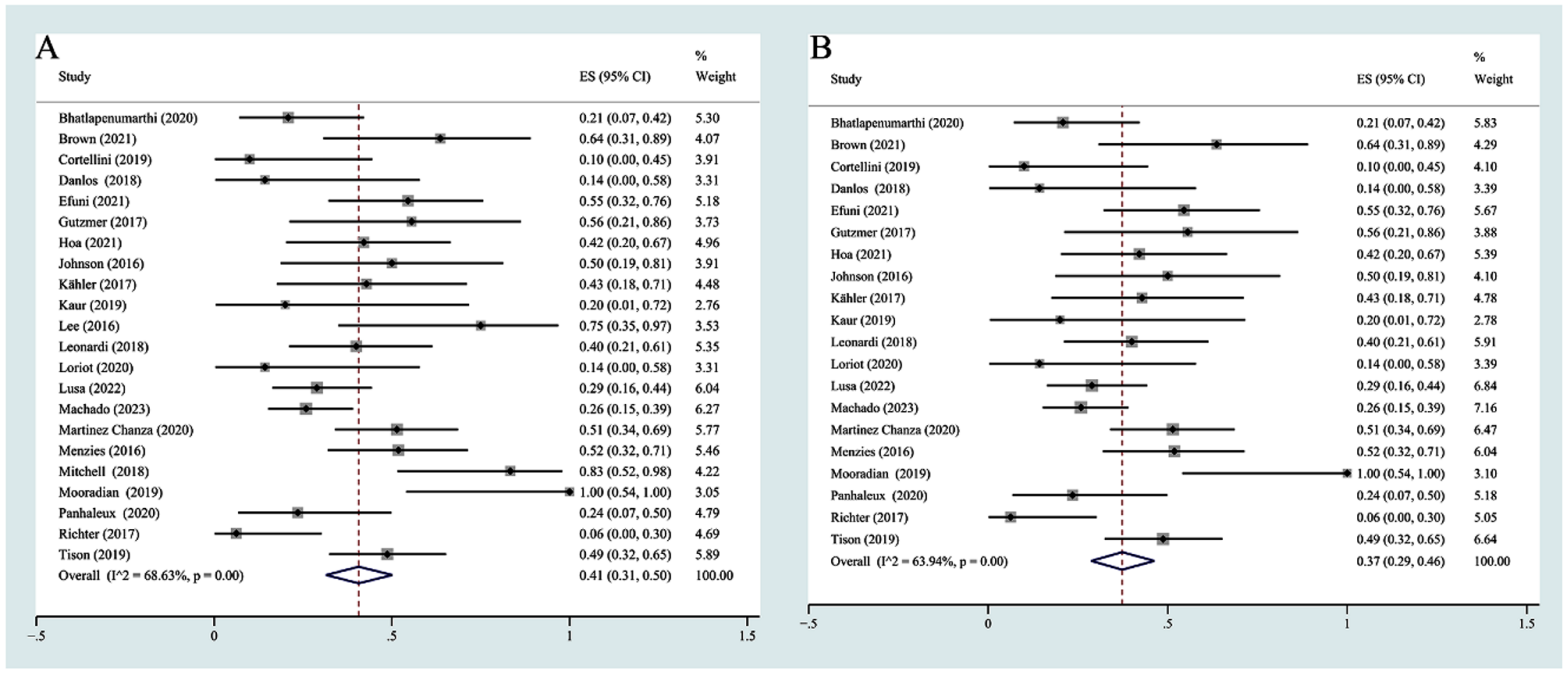
(**A**) The pooled incidence rate of any-grade flares; (**B**) The pooled incidence rate of any-grade flares after excluding the studies from Australia

A total of 22 studies with 426 participants reported flares of rheumatologic disease [17–38]. Due to the high heterogeneity (I^2^ = 68.63%, *P* = 0.00), a random effects model was used for the data analysis, and the combined rate of flares was 41% (95% CI 31%-50%) (Fig. 3A). Considering the surprisingly high incidence of flares from the two Australian studies, we performed an analysis after removing the two studies, and the incidence of flares was 37% (95% CI 20%-46%). Compared with the results before the deletion of the two studies, the incidence of flares decreased by approximately 4% (Fig. 3B). In terms of severity, the incidence of Grade 1–2 flares was 25% (95% CI 14%-38%) (Supplementary Fig. 2A), and the incidence of Grade 3–4 flares was 7% (95% CI 2%-14%) (Supplementary Fig. 2B).

We conducted subgroup analyses stratified by region and type of ICI to determine the underlying heterogeneity. The results of subgroup analysis based on the type of ICI suggested that the incidence of flares in the anti-CTLA-4 therapy group (55% [95% CI 36%-74%) was greater than that in the anti-PD-1/PD-L1 therapy group (31% [95% CI 16%-45%]). The rate of flares differed significantly among the different ICIs (*P* = 0.046). The results of the subgroup analysis based on region suggested that the incidence of flares was 34% (95% CI 20%-49%) in Europe, 35% (95% CI 22%-49%) in North America, 50% (95% CI 38%-61%) in the multination group, and 80% (95% CI 59%-96%) in Australia, with a significant difference among the four groups (*P* = 0.003). In the Australian subgroup, patients were more prone to having underlying rheumatologic disease. The results of subgroup analyses are shown in Supplementary Fig. 3. However, the above subgroups were not significant influencing factors of heterogeneity, which was substantially high (I^2^ > 50%) in all analyses.

Additionally, meta-regression analysis was carried out to investigate the potential source of heterogeneity. The results indicated that the regions (*P* = 0.035) were significantly different and that there was a trend toward significant differences among the study designs (*P* = 0.175), which indicates that regions and study designs may affect heterogeneity. The detailed data are shown in Supplementary Table 5.

#### The incidence of new-onset irAEs

**Fig. 4:**
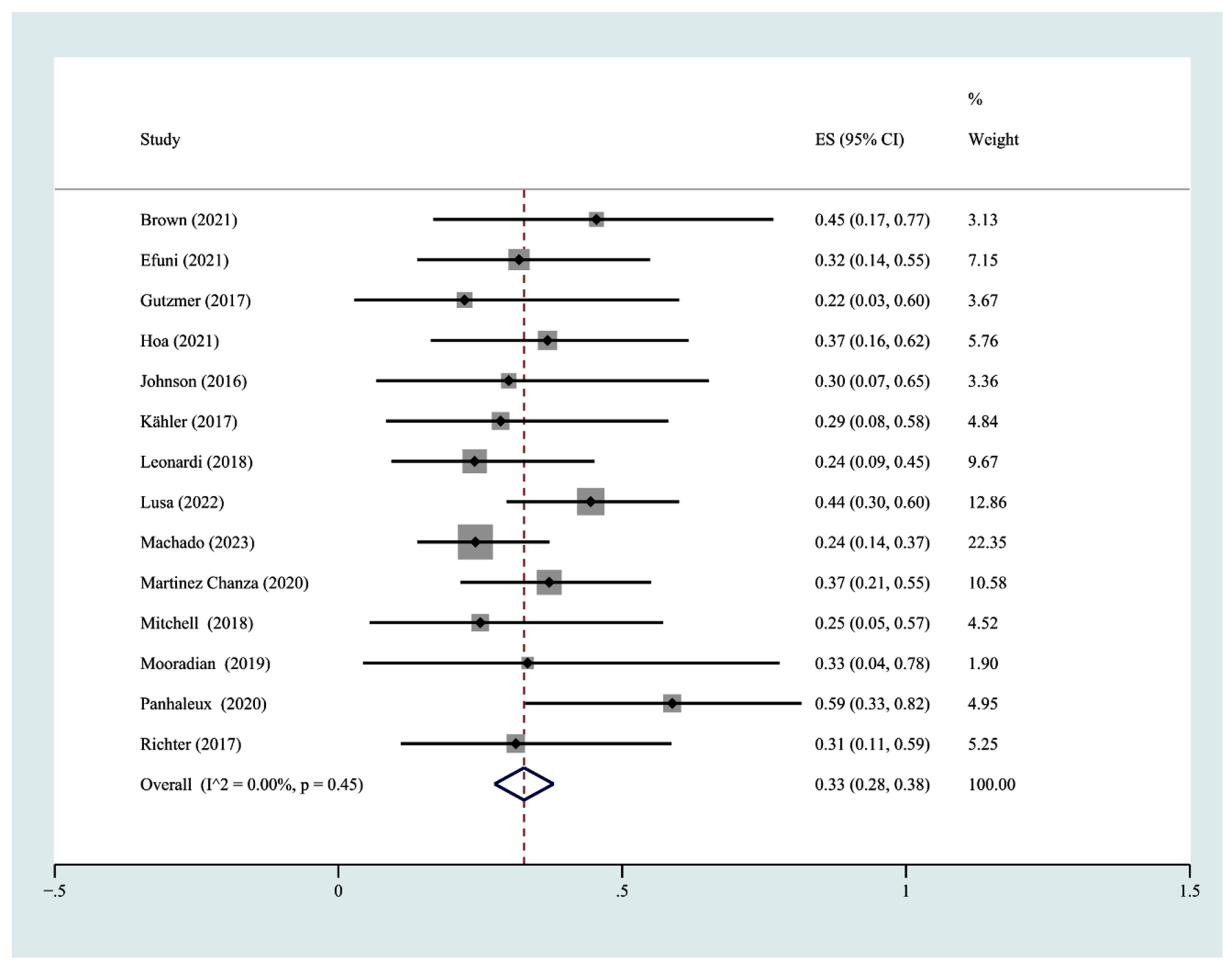
The pooled incidence rate of any-grade new onset irAEs

New-onset irAEs were identified in 14 studies [18, 21–25, 28, 30–32, 34–37] with 299 participants. The incidence of new-onset irAEs ranged from 22.2 to 58.8%, and the pooled incidence of new-onset irAEs was 33% (95% CI 28%-38%, I^2^ = 0) (Fig. 4). In terms of severity, the incidence of Grade 1–2 new-onset irAEs was 19% (95% CI 13%-26%), and the incidence of Grade 3–4 new-onset irAEs was 12% (95% CI 9%-15%). The results are displayed in Supplementary Fig. 2C-D. Furthermore, we conducted subgroup analyses based on region and type of ICI, and no significant differences were observed (Supplementary Fig. 4A-B).

#### Discontinuation due to flares/new onset irAEs

**Fig. 5:**
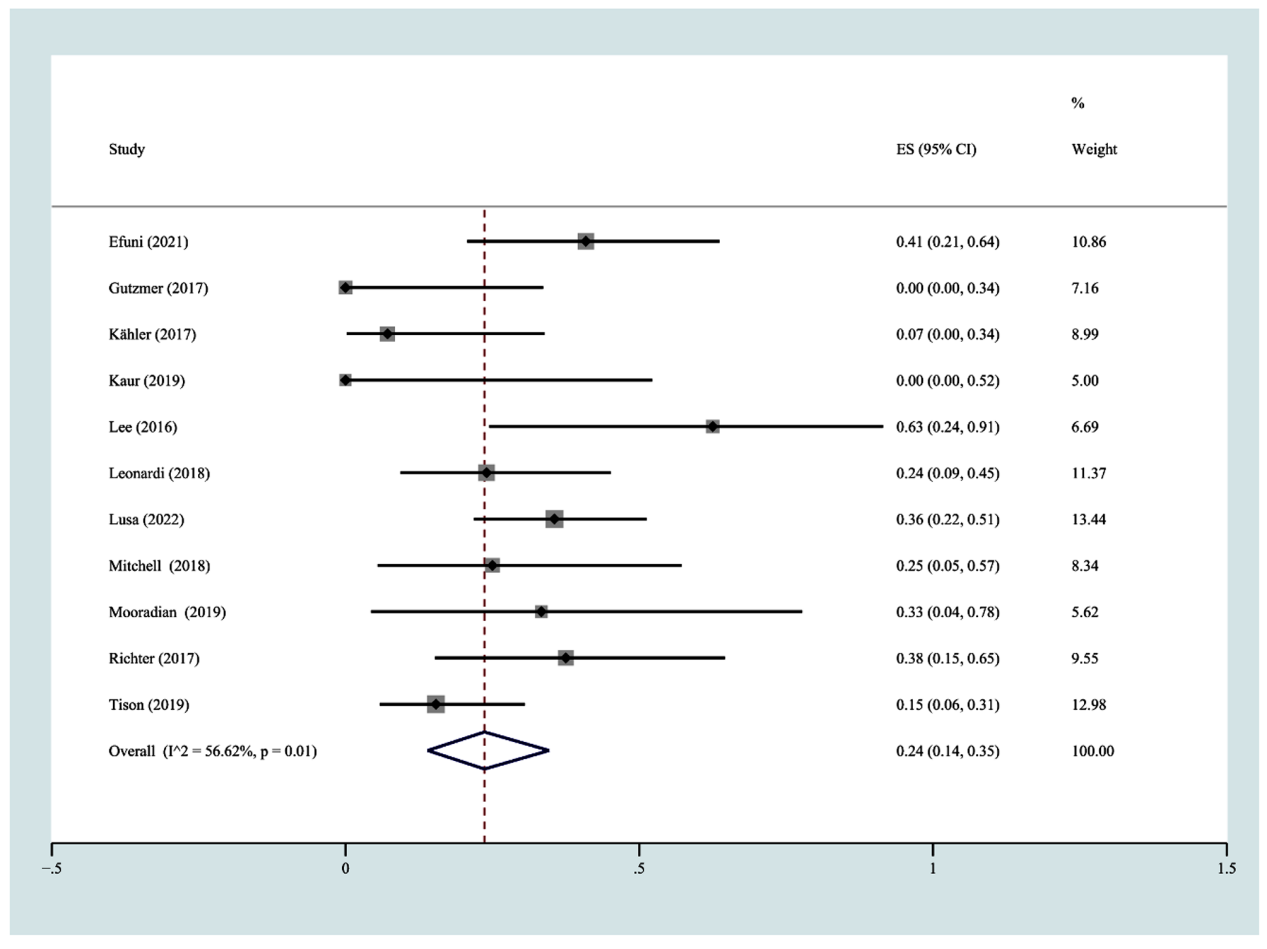
The pooled incidence rate of discontinuation due to flares or new onset irAEs

As shown in Figs. 5 and 11 studies [21, 22, 25–28, 30, 34, 35, 37, 38] with 201 participants reported the discontinuation rate of immunotherapy because of flares or new-onset irAEs. The range of discontinuation rates was 0–62.5%. The incidence of discontinuation was 24% (95% CI 14%-35%) according to a random effects model (I^2^ = 56.62%, *P* = 0.01).

### Clinical efficacy

**Fig. 6:**
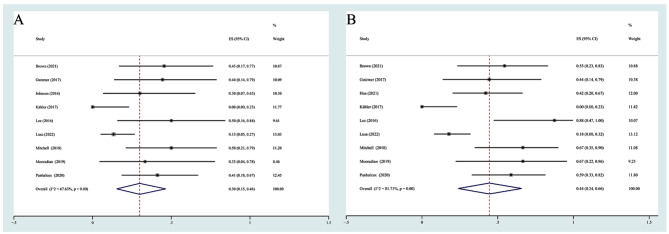
Pooled effect estimates for ICI-treated patients with rheumatologic PAD: (**A**) ORR; (**B**) DCR. ORR: objective response rate; DCR: disease control rate

Among the reported events, 132 participants from nine studies [18, 22, 24, 25, 27, 30, 34–36] reported ORR data. The ORR ranged from 0 to 50%, and the summary ORR was 30% (95% CI 15%-46%) according to a random effects model (I^2^ = 67.63%, *P* = 0.00) (Fig. 6A). Nine studies [18, 22, 23, 25, 27, 30, 34–36] presented data on DCR. The pooled result was 44% (95% CI 24%-66%) based on a random effects model due to significant heterogeneity (I^2^ = 81.71%, *P* = 0.00) (Fig. 6B). We further investigated whether flares had an impact on ORR. We combined the data on the association between flares and ORR from five studies, and the results indicated that the occurrence of flares did not correlate with ORR (RR = 1.10, 95% CI 0.52–2.30) (Supplementary Fig. 5).

### The impact of baseline anti-rheumatic therapy treatment

**Fig. 7:**
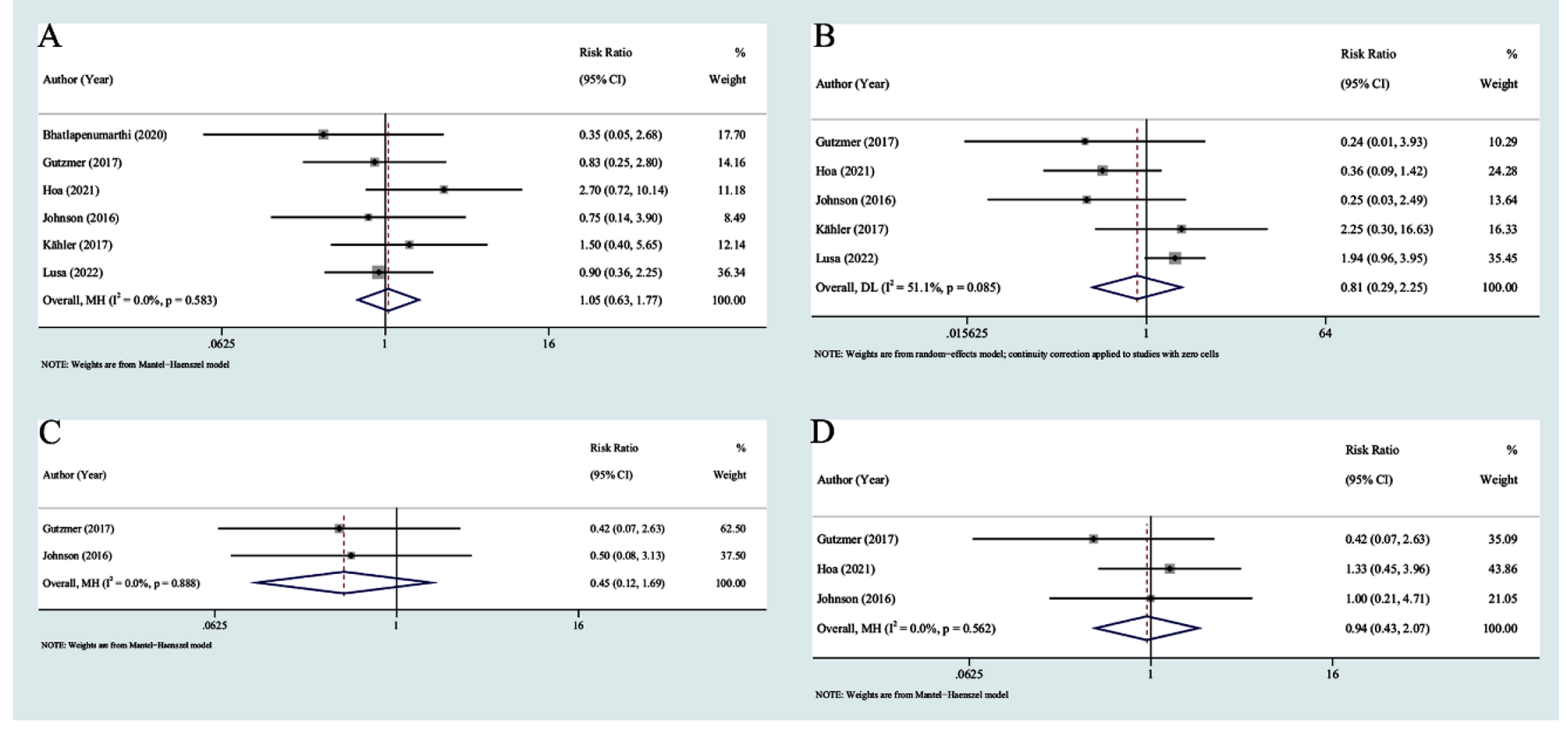
Relative risk in patients with rheumatologic PAD receiving anti-rheumatic treatment at the start of ICI therapy compared with those not receiving treatment: (**A**) flare; (**B**) new-onset irAEs; (**C**) ORR; (**D**) DCR. ORR: objective response rate; DCR: disease control rate

We analyzed 6 studies [17, 22–25, 30] reporting the frequency of flares with and without anti-rheumatic therapy at the start of ICI treatment. Patients who were exposed to anti-rheumatic drugs had a similar risk of flares to those who were not (RR = 1.05, 95% CI 0.63–1.77) (Fig. 7A). We obtained similar findings regarding the association between anti-rheumatic agents and the incidence of new-onset irAEs (RR = 0.81, 95% CI 0.29–2.25) based on 4 studies [22–25, 30] (Fig. 7B). Additionally, the use of anti-rheumatic treatment at the start of ICI therapy was also not associated with the ORR (RR = 0.45, 95% CI 0.12–1.69) or DCR (RR = 0.94, 95% CI 0.43–2.07) (Fig. 7C-D).

### Comparison between RA and other rheumatologic PADs

**Fig. 8:**
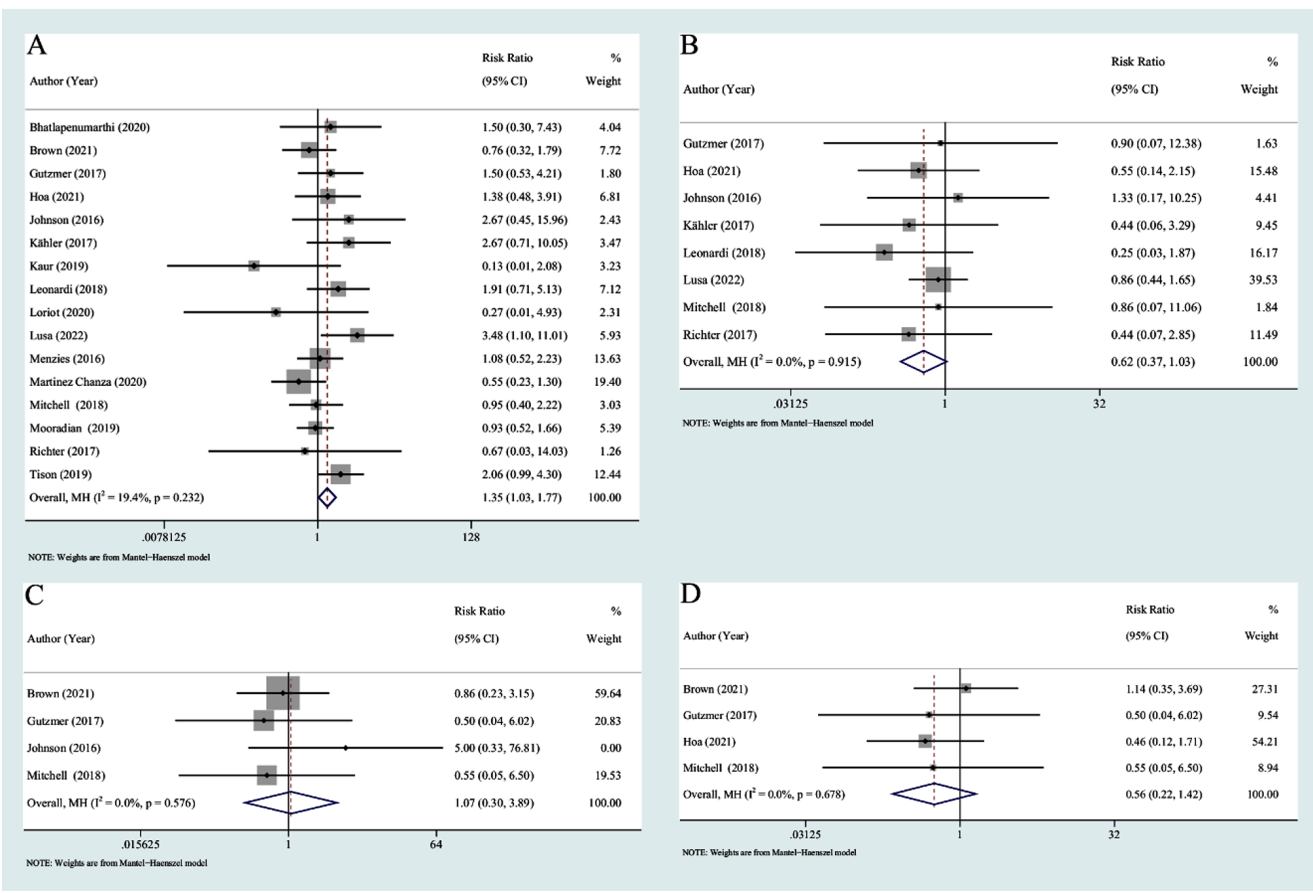
Relative risk in patients with RA compared to those with other autoimmune rheumatologic diseases: (**A**) flare; (**B**) new-onset irAEs; (**C**) ORR; (**D**) DCR. ORR: objective response rate; DCR: disease control rate

RA is the most common subtype of rheumatic disease, and the number of patients with other single rheumatic disease types (such as polymyalgia rheumatica, systemic sclerosis, and Sjogren’s syndrome) extracted from the included studies was low. As a result, we compared the risk of flares between RA and other various autoimmune rheumatologic diseases. A total of 63 out of 134 (47.0%) RA patients had a flare, and 66 out of 170 (38.8%) other autoimmune rheumatologic disease patients developed a flare. The pooled data suggested that patients with RA had a statistically significant greater risk of flares than patients with other rheumatologic PADs did (RR = 1.35, 95% CI 1.03–1.77) (Fig. 8A).

We compared the incidence of new-onset irAEs between patients with RA and patients with other autoimmune rheumatologic diseases. Sixteen out of 60 (26.7%) RA patients experienced new-onset irAEs, and 35 out of 90 (38.8%) other autoimmune rheumatologic disease patients experienced new-onset irAEs. Compared to patients with other autoimmune rheumatologic diseases, patients with RA had a similar risk of new-onset irAEs (RR = 0.62, 95% CI 0.37–1.03) (Fig. 8B).

With respect to the ORR and DCR for different rheumatologic diseases, there was also no significant difference between RA patients and other rheumatologic PAD patients (RR = 1.07, 95% CI = 0.30–3.89 for ORR; RR = 0.56, 95% CI = 0.22–1.42 for DCR) (Fig. 8C and D).

### Publication bias and sensitivity analysis

No obvious publication bias was observed through funnel plots or Begg’s or Egger’s tests (supplementary Fig. 6), indicating the robustness of our study. The sensitivity analyses revealed that no single study could significantly influence our results, which are illustrated in Supplementary Fig. 7.

## Discussion

PD1/PD-L1 and CTLA-4 are the fundamentals of immune regulation [42]. Although ICIs, including antibodies against PD-1/PD-L and CTLA-4, have yielded satisfying outcomes in terms of patient survival, they can also disrupt self-tolerance and lead to unique irAEs [43, 44]. With the widespread application of ICIs in patients with advanced malignant tumors, irAEs caused by ICIs have been adequately discussed in a series of clinical trials [45, 46]. Translational studies have shown that multiple pathways, such as cytokine, autoreactive T-cell and autoantibody pathways, may affect the development of irAEs [42]. However, the safety and efficacy of ICIs in patients with rheumatologic PAD are unknown, as these patients have largely been excluded from clinical trials because of the increased risk of flares. Given the negligible number of rheumatologic PAD patients who may benefit from ICI therapy, an accurate determination of the risk-benefit ratio of ICIs in rheumatologic PAD patients is crucial. To our knowledge, our meta-analysis is the first study to investigate this issue. In this meta-analysis, we conducted a meta-analysis of 23 studies to investigate the impact of rheumatologic PAD on TirAEs, flares, new-onset irAEs and treatment efficacy in patients treated with ICIs. Overall, in this meta-analysis, the pooled incidence of TirAEs was 64% (95% CI 55%-72%). In addition, the incidence of TirAEs was 77% in the anti-CTLA-4 therapy group and 58% in the anti-PD-1/PD-L1 therapy group.

Immune cells in the system affected by PAD are generally abnormally activated, and this system is prone to adverse reactions when patients receive drugs to manipulate the immune environment. In contrast, when the immune cells of other systems unrelated to PAD are in a normal status, the frequency of irAEs is similar to that in the normal population. A recent study from Cai et al. [47] indicated that PAD patients tended to experience irAEs in the same system involved in PAD, whereas the incidence of irAEs concerning other systems that were not affected by PAD was similar to the incidence of irAEs in the non-PAD group. In this meta-analysis, we also reached a similar conclusion: the incidence of flares (41%) was greater than the incidence of new-onset irAEs (33%). The incidences were 7% (95% CI 2%-14%) for Grade 3–4 flares and 12% (95% CI 9%-15%) for Grade 3–4 new-onset irAEs. In addition, 24% of patients discontinued ICIs because of rheumatologic PAD flares or new-onset irAEs. Notably, permanent discontinuation of ICIs may be required because of severe irAEs. Rheumatologic PAD patients require close monitoring for irAEs.

Several previous studies [48, 49] have shown that irAEs caused by ICIs are associated with improved treatment outcomes in cancer patients. Thus, some researchers speculate that PAD patients may benefit more from ICIs due to their tendency toward immune activation. In this meta analysis, the pooled ORR was 30% and DCR was 44% in rheumatologic PAD patients. Additionally, our results showed that flares of potential rheumatologic PAD did not have an association with the ORR (RR = 1.14, 95% CI 0.62–2.08). It is necessary to interpret the results with caution due to selection bias. Patients with a good prognosis may not be treated with ICIs in order to avoid flares of PAD, while patients with a poor prognosis have no choice but to receive ICIs. Due to the heterogeneity of tumor type and tumor stage, we did not further classify the patients into subgroups based on these factors. More prospective studies focused on specific tumor type are required.

A meta-analysis of Xie et al. involving diverse PAD patients revealed that the pooled incidence of flares was 35% (95% CI 29–41%), and compared with other systemic PAD patients, rheumatologic PAD patients had an increased risk of flares; however, these two groups were not significantly different [15]. In the present study, the pooled rate of underlying rheumatologic disorder relapse (41%) was greater than that in the previous meta-analysis. A meta-analysis involving 123 patients also revealed that RA flares were the most common [16]. Previous studies have verified that the expression of PD-1 and PD-L1 is significantly elevated in both early and established RA and that the expression of PD-1 is correlated with the severity of synovial inflammation [50, 51]. In addition, the expression and function of CTLA-4 have been confirmed to be likely related to the pathogenesis of RA [52]. Similarly, the PD-1/PD-L1 and CTLA-4 pathways also play roles in the pathophysiology of other rheumatologic PADs, such as myositis, SLE and Sjögren syndrome [53, 54]. A possible explanation for the greater rate of flares in RA patients was that RA had a stronger connection with the PD-1/PD-L1 and CTLA-4 pathways than other PADs. Another possible explanation was the difference in the use of anti-rheumatic drugs between RA and other rheumatic diseases. Rheumatologists commonly use methotrexate, biologics, and JAK inhibitors for RA, which are typically discontinued when patients are diagnosed with cancers. However, for other rheumatic diseases, glucocorticoids still play a central role. When patients are diagnosed with cancers, glucocorticoids are often continued because they cannot be abruptly discontinued. In this meta-analysis, further exploration of relapses between RA and other subtypes revealed that the rate of flares in RA patients was statistically significant higher than patients with other rheumatologic diseases. Due to the significant heterogeneity in diagnosis of flares identified by different clinicians, the higher rate of flares in the RA population needed to be further confirmed based on individual level data. Interestingly, the rate of new-onset irAEs in RA patients was not greater than that in other rheumatologic PAD patients, which was exactly the opposite of the rate of flares. The contradictory effects of ICIs on flares and new-onset irAEs suggest that there are different mechanisms that lead to the occurrence of flares and new-onset irAEs.

We further conducted subgroup analyses to investigate whether region and ICI type had an impact on the safety of ICIs. We found that regional factors may influence drug safety assessments. The incidence of TirAEs and flares in the Australian population was significantly greater than that in other regions. Patients from different regions may have various ethnic backgrounds, disparate treatment standards, different geographic environments, and CTLA-4 gene polymorphisms [53]. These factors may lead to differences in clinical outcomes. In addition, after removing the two Australian studies, the pooled results were only slightly lower than before. The small sample size and low weight of the Australian studies could explain this phenomenon. In the subgroup analysis of new-onset irAEs stratified by region, only one study was included in Australia, so no significant results were obtained. With knowledge of this difference, further race-conscious research is urgently needed to confirm the association between geographical region and the safety of ICIs.

Regarding the types of ICIs, flares occurred more often in the anti-CTLA-4 therapy group than in the anti-PD-1/PD-L1 therapy group. We also found a similar trend in the subgroup analysis of the TirAEs. These findings was consistent with the conclusion that the risk of any grade of irAEs was greater in the anti-CTLA-4 therapy group than in the anti-PD-1/PD-L1 therapy group in the general cancer population [55]. However, another meta-analysis conducted by Abdel-Wahab et al. reached the contradictory conclusion that the rate of flares in patients receiving anti-PD-1 therapy was greater than that in patients receiving anti-CTLA-4 therapy [16]. This disparate result may be due to the difference in the types of autoimmune diseases between our meta-analysis and previous meta-analysis and the small sample size of the previous study. Another potential confounding factor for these results was the imbalance in terms of the number of patients receiving different ICI regimens in this meta-analysis. There were 11 studies that included 159 patients who received anti-PD-1/PD-L1 therapy, while there were only 3 studies that included 32 patients who received anti-CTLA-4 therapy. The number of patients receiving anti-PD-1/PD-L1 monotherapy was significantly greater than that receiving anti-CTLA-4 monotherapy. The fact that treatment regimens were not balanced in rheumatologic PAD patients may have affected the results.

Horvat et al. reported that the use of corticosteroids for the treatment of irAEs in cancer patients was not associated with the efficacy of ICIs [56]. In this meta-analysis, we examined the association between the use of baseline anti-rheumatic therapy for rheumatic disease and patient outcomes. The results suggested that the incidence of flares, incidence of new-onset irAEs and ORR in patients with baseline anti-rheumatic therapy at the start of treatment were similar to those in patients without anti-rheumatic therapy, in line with prior studies focused on patients with diverse PADs [28, 30]. However, Arbor et al. [57] reported that the use of baseline corticosteroids had a negative influence on the efficacy of ICIs. The underlying mechanism may be that baseline corticosteroid treatment weakened the proliferative burst of CD8-positive T cells required in response to ICIs. The impact of anti-rheumatic therapy on irAEs and outcomes may be different for patients receiving different types of anti-rheumatic therapy for their rheumatologic diseases. However, because the anti-rheumatic therapy regimens used in each study were not uniform and included hydroxychloroquine, methotrexate, prednisone, azathioprine, etanercept, and sulfasalazine, we did not stratify patients according to the type of anti-rheumatic therapy. This phenomenon needs to be further studied in future research. Notably, due to retrospective nature of the most included studies, there was inherent selection bias in evaluating the impact of baseline anti-rheumatic treatment. Patients with higher risk of flares or active rheumatologic PAD were more likely to require anti-rheumatic therapy.

There are also several factors that may affect the safety and effectiveness of ICIs in rheumatologic PAD patients. First, patients who have clinically active disease or more severe disease at the time of ICI therapy may have a greater rate of flares [18, 28]. However, only two articles have reported whether rheumatologic PAD patients have active symptoms, making comparisons of associations between different activities at baseline and outcomes difficult. Second, the incidence of irAEs may be affected by treatment strategies. A previous meta-analysis suggested that ICIs combined with chemotherapy reduce the incidence of irAEs in advanced NSCLC patients compared with that of ICI monotherapy, possibly due to the immunosuppressive effect of chemotherapy. Bone marrow suppression by chemotherapy may limit immune system overactivation [58]. There was no detailed explanation of whether systemic chemotherapy was administered prior to ICI therapy or during ICI therapy in the included studies. Therefore, subgroup analysis based on treatment strategies could not be carried out. In addition, due to a lack of data, the impacts of sex, stage, line of ICI treatment, presence of PAD-related autoantibodies, ICIs type, drug dose, underlying disease type, underlying disease activity, comfort of treating physicians, patient preference and timing of cancer diagnosis on outcomes were not assessed. Discussing the association between these potential influencing factors and irAEs will be the research area of future studies.

To date, our study is the first meta-analysis to evaluate the safety and efficacy of ICIs in patients with rheumatologic PAD and fill gaps in knowledge. Unlike previous studies, this study specifically focused on individuals with rheumatologic PAD rather than individuals with various immune diseases. Our study can reduce heterogeneity caused by PADs in different systems. Immune-mediated endocrine dysfunction, such as thyroid dysfunction, is one of the most common irAEs and does not hinder further treatment with ICIs [59]. If the PAD included the endocrine PAD patient subtypes, there was a confounding effect on the results [60]. In addition, we further discussed the impact of risk factors such as region, type of ICI, and rheumatologic disease subtype on the safety and effectiveness of ICIs and provided a reference for clinical physicians to identify high-risk populations.

However, there are certain limitations to the present study. First, almost all the included studies were single-arm observational studies, and patient information was obtained from the patients’ medical records. In such studies, incomplete reports may lead to an underestimation of the incidence of irAEs. Second, the largest confounding factor was not addressed in the included studies. There are no unified standards in terms of patients with PAD who were offered ICIs. Compared to patients with active disease requiring immunosuppressants and with better prognosis cancers, clinicians prefer to use ICIs in patients who are inactive and not receiving immunosuppressive therapy and who have a worse prognosis. Most included patients had quiescent disease and inactive symptoms in some studies, while patients with more severe rheumatic disease were under-represented, resulting in selection bias in this meta-analysis. Third, there was another large confounding by indication bias in our meta-analysis. Some authors of the included studies were rheumatologists, and for patients with less severe disease who were not referred for rheumatologic assessment and were managed by the oncology team, such patients may not be included. Only patients with symptoms significant enough to be referred for the assistance of rheumatologists were reported by rheumatologists. Finally, the final sample size was relatively small, although all studies reporting the safety and effectiveness of ICIs for the treatment of rheumatologic PAD were included, which may limit the generalizability of our results. More large-scale prospective studies are necessary to improve the level of evidence.

## Conclusions

Patients with rheumatologic PAD, particularly those with RA, appear susceptible to relapses of their underlying disease following ICI therapy. However, ICIs were efficacious for cancer patients with rheumatologic PAD. The frequency of flares significantly differed according to the type of ICI and region. Rheumatologic PAD is not an absolute contraindication for ICIs. Physicians should carefully assess the individual risk of each patient based on the type, activity, and severity of PAD. Close monitoring and timely management by a multidisciplinary team are paramount for patients with an elevated risk of PAD flares. Further large-scale prospective studies adjusting for various confounding factors are essential to validate these results and identify predictive markers for irAEs and efficacy in rheumatologic PAD patients.

### Electronic supplementary material

Below is the link to the electronic supplementary material.

Supplementary Material 1

Supplementary Material 2

Supplementary Material 3

Supplementary Material 4

Supplementary Material 5

Supplementary Material 6

Supplementary Material 7

Supplementary Material 8

Supplementary Material 9

Supplementary Material 10

Supplementary Material 11

Supplementary Material 12

## Data Availability

Data is provided within the manuscript or supplementary information files.

## Abbreviations

ICIs: Immune checkpoint inhibitors
PAD: Preexisting autoimmune disease
CTLA-4: Cytotoxic T-lymphocyte-associated-4
PD-1: Programmed cell death 1
PD-L1: Programmed death-ligand 1
irAEs: Immune-related adverse events
TirAEs: Either flare, new onset irAEs or both
RA: Rheumatoid arthritis
ORR: Objective response rate
DCR: Disease control rate

## References

[1] Motzer RJ, Escudier B, McDermott DF, George S, Hammers HJ, Srinivas S (2015). Nivolumab versus Everolimus in Advanced Renal-Cell Carcinoma. N Engl J Med.

[2] Wolchok JD, Chiarion-Sileni V, Gonzalez R, Rutkowski P, Grob JJ, Cowey CL (2017). Overall survival with combined Nivolumab and Ipilimumab in Advanced Melanoma. N Engl J Med.

[3] Reck M, Rodríguez-Abreu D, Robinson AG, Hui R, Csőszi T, Fülöp A (2016). Pembrolizumab versus Chemotherapy for PD-L1-Positive non-small-cell Lung Cancer. N Engl J Med.

[4] Tarhini A, Lo E, Minor DR (2010). Releasing the brake on the immune system: ipilimumab in melanoma and other tumors. Cancer Biother Radiopharm.

[5] Brahmer JR, Lacchetti C, Schneider BJ, Atkins MB, Brassil KJ, Caterino JM (2018). Management of Immune-related adverse events in patients treated with Immune checkpoint inhibitor therapy: American Society of Clinical Oncology Clinical Practice Guideline. J Clin Oncology: Official J Am Soc Clin Oncol.

[6] Postow MA, Sidlow R, Hellmann MD (2018). Immune-related adverse events Associated with Immune Checkpoint Blockade. N Engl J Med.

[7] Simon TA, Thompson A, Gandhi KK, Hochberg MC, Suissa S (2015). Incidence of malignancy in adult patients with rheumatoid arthritis: a meta-analysis. Arthritis Res Therapy.

[8] Olesen AB, Svaerke C, Farkas DK, Sørensen HT (2010). Systemic sclerosis and the risk of cancer: a nationwide population-based cohort study. Br J Dermatol.

[9] Liang Y, Yang Z, Qin B, Zhong R (2014). Primary Sjogren’s syndrome and malignancy risk: a systematic review and meta-analysis. Ann Rheum Dis.

[10] Khan SA, Pruitt SL, Xuan L, Gerber DE (2016). Prevalence of Autoimmune Disease among patients with Lung Cancer: implications for Immunotherapy Treatment options. JAMA Oncol.

[11] Sandigursky S, Silverman GJ, Mor A (2017). Targeting the programmed cell death-1 pathway in rheumatoid arthritis. Autoimmun Rev.

[12] Hossen MM, Ma Y, Yin Z, Xia Y, Du J, Huang JY (2023). Current understanding of CTLA-4: from mechanism to autoimmune diseases. Front Immunol.

[13] Zhang Q, Vignali DA (2016). Co-stimulatory and co-inhibitory pathways in autoimmunity. Immunity.

[14] Cai Q, Huo G-w, Zhu F-y, Yue P, Yuan D-q, Chen P. Safety and efficacy of immune checkpoint inhibitors in advanced cancer patients with autoimmune disease: a meta-analysis. Hum Vaccines Immunotherapeutics. 2022;18(7). 10.1080/21645515.2022.2145102 PMC9762847 36471629

[15] Xie W, Huang H, Xiao S, Fan Y, Deng X, Zhang Z (2020). Immune checkpoint inhibitors therapies in patients with cancer and preexisting autoimmune diseases: a meta-analysis of observational studies. Autoimmun rev.

[16] Abdel-Wahab N, Shah M, Lopez-Olivo MA, Suarez-Almazor ME (2018). Use of Immune checkpoint inhibitors in the treatment of patients with Cancer and Preexisting Autoimmune Disease: a systematic review. Ann Intern Med.

[17] Bhatlapenumarthi V, Patwari A, Harb AJ (2021). Immune-related adverse events and immune checkpoint inhibitor tolerance on rechallenge in patients with irAEs: a single-center experience. J Cancer Res Clin Oncol.

[18] Brown LJ, Weppler A, Bhave P, Allayous C, Patrinely JR, Ott P (2021). Combination anti-PD1 and ipilimumab therapy in patients with advanced melanoma and pre-existing autoimmune disorders. J Immunother Cancer.

[19] Cortellini A, Buti S, Santini D, Perrone F, Giusti R, Tiseo M (2019). Clinical outcomes of patients with Advanced Cancer and Pre-existing Autoimmune diseases treated with anti-programmed Death-1 immunotherapy: a real-world transverse study. Oncologist.

[20] Danlos F-X, Voisin A-L, Dyevre V, Michot J-M, Routier E, Taillade L (2018). Safety and efficacy of anti-programmed death 1 antibodies in patients with cancer and pre-existing autoimmune or inflammatory disease. Eur J Cancer.

[21] Efuni E, Cytryn S, Boland P, Niewold TB, Pavlick A, Weber J et al. Risk of toxicity after initiating immune checkpoint inhibitor treatment in patients with rheumatoid arthritis. JCR: Journal of Clinical Rheumatology. 2021;27(7):267– 71. 10.1097/RHU.0000000000001314 PMC7374048 31977647

[22] Gutzmer R, Koop A, Meier F, Hassel JC, Terheyden P, Zimmer L (2017). Programmed cell death protein-1 (PD-1) inhibitor therapy in patients with advanced melanoma and preexisting autoimmunity or ipilimumab-triggered autoimmunity. Eur J Cancer.

[23] Hoa S, Laaouad L, Roberts J, Ennis D, Ye C, Al Jumaily K (2021). Preexisting autoimmune disease and immune-related adverse events associated with anti-PD-1 cancer immunotherapy: a national case series from the Canadian Research Group of Rheumatology in Immuno-Oncology. Cancer Immunol Immunother.

[24] Johnson DB, Sullivan RJ, Ott PA, Carlino MS, Khushalani NI, Ye F (2016). Ipilimumab Therapy in patients with Advanced Melanoma and Preexisting Autoimmune disorders. JAMA Oncol.

[25] Kähler KC, Eigentler TK, Gesierich A, Heinzerling L, Loquai C, Meier F (2018). Ipilimumab in metastatic melanoma patients with pre-existing autoimmune disorders. Cancer Immunol Immunother.

[26] Kaur A, Doberstein T, Amberker RR, Garje R, Field EH, Singh N (2019). Immune-related adverse events in cancer patients treated with immune checkpoint inhibitors. Medicine.

[27] Lee B, Wong A, Kee D, Neeson P, Shackleton M, McArthur G (2016). The use of ipilimumab in patients with rheumatoid arthritis and metastatic melanoma. Annals Oncology: Official J Eur Soc Med Oncol.

[28] Leonardi GC, Gainor JF, Altan M, Kravets S, Dahlberg SE, Gedmintas L (2018). Safety of programmed Death-1 pathway inhibitors among patients with non-small-cell Lung Cancer and Preexisting Autoimmune disorders. J Clin Oncol.

[29] Loriot Y, Sternberg CN, Castellano D, Oosting SF, Dumez H, Huddart R (2020). Safety and efficacy of atezolizumab in patients with autoimmune disease: subgroup analysis of the SAUL study in locally advanced/metastatic urinary tract carcinoma. Eur J Cancer.

[30] Lusa A, Alvarez C, Saxena Beem S, Schwartz TA, Ishizawar R. Immune-related adverse events in patients with pre-existing autoimmune rheumatologic disease on immune checkpoint inhibitor therapy. BMC Rheumatol. 2022;6(1). 10.1186/s41927-022-00297-5 PMC9641936 36345032

[31] Machado A, Shatila M, Liu C, Wang J, Altan M, Zhang HC (2023). Immune-related adverse events after immune checkpoint inhibitor exposure in adult cancer patients with pre-existing autoimmune diseases. J Cancer Res Clin Oncol.

[32] Martinez Chanza N, Xie W, Issa M, Dzimitrowicz H, Tripathi A. Safety and efficacy of immune checkpoint inhibitors in advanced urological cancers with pre-existing autoimmune disorders: a retrospective international multicenter study. J Immunother Cancer. 2020;8(1). 10.1136/jitc-2020-000538 PMC7174076 32217762

[33] Menzies AM, Johnson DB, Ramanujam S, Atkinson VG, Wong ANM, Park JJ (2017). Anti-PD-1 therapy in patients with advanced melanoma and preexisting autoimmune disorders or major toxicity with ipilimumab. Ann Oncol.

[34] Mitchell EL, Lau PKH, Khoo C, Liew D, Leung J, Liu B (2018). Rheumatic immune-related adverse events secondary to anti–programmed death-1 antibodies and preliminary analysis on the impact of corticosteroids on anti-tumour response: a case series. Eur J Cancer.

[35] Mooradian MJ, Nasrallah M, Gainor JF, Reynolds KL, Cohen JV, Lawrence DP (2019). Musculoskeletal rheumatic complications of immune checkpoint inhibitor therapy: a single center experience. Semin Arthritis Rheum.

[36] Panhaleux M, Espitia O, Terrier B, Manson G, Maria A, Humbert S (2022). Anti–programmed death ligand 1 immunotherapies in cancer patients with pre-existing systemic sclerosis: a postmarketed phase IV safety assessment study. Eur J Cancer.

[37] Richter MD, Pinkston O, Kottschade LA, Finnes HD, Markovic SN, Thanarajasingam U (2018). Brief report: Cancer immunotherapy in patients with Preexisting Rheumatic Disease: the Mayo Clinic Experience. Arthritis Rheumatol.

[38] Tison A, Quéré G, Misery L, Funck-Brentano E, Danlos FX, Routier E (2019). Safety and Efficacy of Immune checkpoint inhibitors in patients with Cancer and Preexisting Autoimmune Disease: a Nationwide, Multicenter Cohort Study. Arthritis Rheumatol.

[39] van der Kooij MK, Suijkerbuijk KPM, Aarts MJB, van den Berkmortel FWPJ, Blank CU, Boers-Sonderen MJ (2021). Safety and efficacy of checkpoint inhibition in patients with Melanoma and Preexisting Autoimmune Disease. Ann Intern Med.

[40] Stang A (2010). Critical evaluation of the Newcastle-Ottawa scale for the assessment of the quality of nonrandomized studies in meta-analyses. Eur J Epidemiol.

[41] Munn Z, Barker TH, Moola S, Tufanaru C, Stern C, McArthur A (2020). Methodological quality of case series studies: an introduction to the JBI critical appraisal tool. JBI Evid Synthesis.

[42] Kennedy LB, Salama AKS (2020). A review of cancer immunotherapy toxicity. Cancer J Clin.

[43] Haanen J, Ernstoff MS, Wang Y, Menzies AM, Puzanov I, Grivas P (2020). Autoimmune diseases and immune-checkpoint inhibitors for cancer therapy: review of the literature and personalized risk-based prevention strategy. Ann Oncol.

[44] Kroschinsky F, Stölzel F, von Bonin S, Beutel G, Kochanek M, Kiehl M (2017). New drugs, new toxicities: severe side effects of modern targeted and immunotherapy of cancer and their management. Crit Care (London England).

[45] Weber JS, Dummer R, de Pril V, Lebbé C, Hodi FS (2013). Patterns of onset and resolution of immune-related adverse events of special interest with ipilimumab: detailed safety analysis from a phase 3 trial in patients with advanced melanoma. Cancer.

[46] Metro G, Ricciuti B, Brambilla M, Baglivo S, Soli I, Minenza E (2017). The safety of nivolumab for the treatment of advanced non-small cell lung cancer. Exp Opin Drug Saf.

[47] Cai Q, Huo GW, Zhu FY, Yue P, Yuan DQ, Chen P (2022). Safety and efficacy of immune checkpoint inhibitors in advanced cancer patients with autoimmune disease: a meta-analysis. Hum Vaccin Immunother.

[48] Freeman-Keller M, Kim Y, Cronin H, Richards A, Gibney G, Weber JS (2016). Nivolumab in Resected and Unresectable Metastatic Melanoma: characteristics of Immune-Related Adverse Events and Association with outcomes. Clin cancer Research: Official J Am Association Cancer Res.

[49] Haratani K, Hayashi H, Chiba Y, Kudo K, Yonesaka K, Kato R (2018). Association of Immune-related adverse events with Nivolumab Efficacy in Non-small-cell Lung Cancer. JAMA Oncol.

[50] Canavan M, Floudas A, Veale DJ, Fearon U (2021). The PD-1:PD-L1 axis in inflammatory arthritis. BMC Rheumatol.

[51] Raptopoulou AP, Bertsias G, Makrygiannakis D, Verginis P, Kritikos I, Tzardi M (2010). The programmed death 1/programmed death ligand 1 inhibitory pathway is up-regulated in rheumatoid synovium and regulates peripheral T cell responses in human and murine arthritis. Arthritis Rheum.

[52] Körmendy D, Hoff H, Hoff P, Bröker BM, Burmester GR, Brunner-Weinzierl MC (2013). Impact of the CTLA-4/CD28 axis on the processes of joint inflammation in rheumatoid arthritis. Arthritis Rheum.

[53] Zhou C, Gao S, Yuan X, Shu Z, Li S, Sun X (2021). Association between CTLA-4 gene polymorphism and risk of rheumatoid arthritis: a meta-analysis. Aging.

[54] Kobayashi M, Kawano S, Hatachi S, Kurimoto C, Okazaki T, Iwai Y (2005). Enhanced expression of programmed death-1 (PD-1)/PD-L1 in salivary glands of patients with Sjögren’s syndrome. J Rhuematol.

[55] El Osta B, Hu F, Sadek R, Chintalapally R, Tang SC (2017). Not all immune-checkpoint inhibitors are created equal: Meta-analysis and systematic review of immune-related adverse events in cancer trials. Crit Rev Oncol/Hematol.

[56] Horvat TZ, Adel NG, Dang TO, Momtaz P, Postow MA, Callahan MK (2015). Immune-related adverse events, need for systemic immunosuppression, and effects on Survival and Time to Treatment failure in patients with melanoma treated with Ipilimumab at Memorial Sloan Kettering Cancer Center. J Clin Oncol.

[57] Arbour KC, Mezquita L, Long N, Rizvi H, Auclin E, Ni A (2018). Impact of baseline steroids on efficacy of programmed cell Death-1 and programmed death-ligand 1 blockade in patients with non-small-cell Lung Cancer. J Clin Oncology: Official J Am Soc Clin Oncol.

[58] Wang M, Liang H (2021). Immune-related adverse events of a PD-L1 inhibitor plus chemotherapy versus a PD-L1 inhibitor alone in first-line treatment for advanced non-small cell lung cancer: a meta-analysis of randomized control trials. Cancer.

[59] Cooksley T, Girotra M, Ginex P, Gordon RA, Anderson R, Blidner A (2020). Multinational Association of Supportive Care in Cancer (MASCC) 2020 clinical practice recommendations for the management of immune checkpoint inhibitor endocrinopathies and the role of advanced practice providers in the management of immune-mediated toxicities. Support Care Cancer.

[60] Yamaguchi A, Saito Y, Okamoto K, Narumi K, Furugen A, Takekuma Y (2021). Preexisting autoimmune disease is a risk factor for immune-related adverse events: a meta-analysis. Support Care Cancer.

