# Supplementary material for: Immune checkpoint inhibitors in Cancer patients with rheumatologic preexisting autoimmune diseases: a systematic review and meta-analysis
Source: BMC Cancer. 2024 Apr 17;24:490. doi: 10.1186/s12885-024-12256-z (PMC11025164; doi:10.1186/s12885-024-12256-z)
Supplement: Supplementary file 1 — Supplementary Material 1 [file 12885_2024_12256_MOESM1_ESM.docx]

**Supplementary Table 1.** Search strategy

| **PubMed** | **Searching Strategy** | **Results** |
| --- | --- | --- |
| #1 | "Neoplasms"[MeSH Terms] OR "Tumor"[Title/Abstract] OR "Neoplasm"[Title/Abstract] OR "Tumors"[Title/Abstract] OR "Neoplasia"[Title/Abstract] OR "Neoplasias"[Title/Abstract] OR "Cancer"[Title/Abstract] OR "Cancers"[Title/Abstract] OR "malignant neoplasm"[Title/Abstract] OR "Malignancy"[Title/Abstract] OR "Malignancies"[Title/Abstract] OR "malignant neoplasms"[Title/Abstract] OR "neoplasm malignant"[Title/Abstract] OR "neoplasms malignant"[Title/Abstract] OR "benign neoplasms"[Title/Abstract] OR "benign neoplasm"[Title/Abstract] OR "neoplasms benign"[Title/Abstract] OR "neoplasm benign"[Title/Abstract] | 4875399 |
| #2 | "Immune Checkpoint Inhibitors"[Mesh] OR "Immune Checkpoint Inhibitor"[Title/Abstract] OR "Immune Checkpoint Blocker"[Title/Abstract] OR "Immune Checkpoint Blockade"[Title/Abstract] OR "Immune Checkpoint Inhibition"[Title/Abstract] OR "PD-L1 Inhibitor*"[Title/Abstract] OR "Programmed Death Ligand 1 Inhibitor"[Title/Abstract] OR "PD-1-PD-L1 Blockade"[Title/Abstract] OR "CTLA-4 Inhibitor"[Title/Abstract] OR "Cytotoxic T Lymphocyte Associated Protein 4 Inhibitor"[Title/Abstract] OR "PD-1 Inhibitor"[Title/Abstract] OR "Programmed Cell Death Protein 1 Inhibitor"[Title/Abstract] OR "nivolumab"[Title/Abstract] OR "pembrolizumab"[Title/Abstract] OR"atezolizumab"[Title/Abstract] OR "camrelizumab"[Title/Abstract] OR "cemiplimab"[Title/Abstract] OR "durvalumab"[Title/Abstract] OR "toripalimab"[Title/Abstract] OR "tislelizumab"[Title/Abstract] OR “Penpulimab "[Title/Abstract] OR "Sintilimab "[Title/Abstract] OR "zimberelimab"[Title/Abstract] OR " Sugemalimab"[Title/Abstract] | 31603 |
| #3 | "Rheumatic Diseases"[MeSH Terms] OR "Rheumatic"[Title/Abstract] OR "Rheumatic Diseases"[Title/Abstract] OR "rheumatics"[Title/Abstract] OR "Rheumatism"[Title/Abstract] OR "rheumatisms"[Title/Abstract] OR "Pseudopolyarthritis"[Title/Abstract] OR "Rhizomelic"[Title/Abstract] OR "Rhizomelic"[Title/Abstract] OR "rhizomelic pseudopolyarthritis"[Title/Abstract] OR "arthritis, rheumatoid"[MeSH Terms] OR "rheumatoid arthritis"[Title/Abstract] OR "Collagen Diseases"[MeSH Terms] OR "collagen disease"[Title/Abstract] OR "disease collagen"[Title/Abstract] OR "Uveitis"[MeSH Terms] OR "Connective Tissue Diseases"[MeSH Terms] OR "Uveitides"[Title/Abstract] OR "connective tissue disease"[Title/Abstract] OR "disease connective tissue"[Title/Abstract] OR "diseases connective tissue"[Title/Abstract] OR "Giant Cell Arteritis"[MeSH Terms] OR "Arteritis"[MeSH Terms] OR "Arteritis"[Title/Abstract] OR "Arteritides"[Title/Abstract] OR "arteritis giant cell"[Title/Abstract] OR "giant cell arteritides"[Title/Abstract] OR "Giant Cell Arteritis"[Title/Abstract] OR "arteritis giant cell"[Title/Abstract] OR "horton giant cell arteritis"[Title/Abstract] OR "Churg-Strauss Syndrome"[MeSH Terms] OR "Churg-Strauss Syndrome"[Title/Abstract] OR "syndrome churg strauss"[Title/Abstract] OR "allergic granulomatous angiitis"[Title/Abstract] OR "Vasculitis"[MeSH Terms] OR "Vasculitis"[Title/Abstract] OR "CREST Syndrome"[MeSH Terms] OR "crest syndromes"[Title/Abstract] OR "syndrome crest"[Title/Abstract] OR "raynaud disease"[MeSH Terms] OR "raynaud"[Title/Abstract] OR "raynaud disease"[Title/Abstract] OR "raynauds"[Title/Abstract] OR "Raynaud's"[Title/Abstract] OR "Calcinosis-Raynaud"[Title/Abstract] OR "Spondylarthritis"[MeSH Terms] OR "Spondylarthritides"[Title/Abstract] OR "spinal arthritis"[Title/Abstract] OR "polyarthritides"[Title/Abstract] OR "spondyloarthritis ankylopoietica"[Title/Abstract] OR "ankylosing spondylitis"[Title/Abstract] OR "ankylosing spondylarthritis"[Title/Abstract] OR "ankylosing spondylarthritides"[Title/Abstract] OR "spondylarthritides ankylosing"[Title/Abstract] OR "spondylarthritis ankylosing"[Title/Abstract] OR "Spondyloarthritides"[Title/Abstract] OR "rheumatoid spondylitis"[Title/Abstract] OR "spondylitis rheumatoid"[Title/Abstract] OR "Myositis"[MeSH Terms] OR "Myositides"[Title/Abstract] OR "myopathy inflammatory"[Title/Abstract] OR "muscle diseases inflammatory"[Title/Abstract] OR "inflammatory muscle diseases"[Title/Abstract] OR "inflammatory muscle disease"[Title/Abstract] OR "inflammatory myopathies"[Title/Abstract] OR "myopathies inflammatory"[Title/Abstract] OR "Sjogren's Syndrome"[MeSH Terms] OR "sjogrens syndrome"[Title/Abstract] OR "syndrome sjogren s"[Title/Abstract] OR "sjogren syndrome"[Title/Abstract] OR "scleroderma, systemic"[MeSH Terms] OR "systemic sclerosis"[Title/Abstract] OR "sclerosis systemic"[Title/Abstract] OR "systemic scleroderma"[Title/Abstract] OR "Scleroderma"[Title/Abstract] OR "lupus erythematosus, systemic"[MeSH Terms] OR "systemic lupus erythematosus"[Title/Abstract] OR "lupus erythematosus disseminatus"[Title/Abstract] OR "Sarcoidosis"[MeSH Terms] OR "Sarcoidoses"[Title/Abstract] OR "boeck sarcoid"[Title/Abstract] OR "boecks sarcoid"[Title/Abstract] OR "sarcoidal"[Title/Abstract] OR psoriatic"[MeSH Terms] OR "psoriasis arthritic"[Title/Abstract] OR "arthritic psoriasis"[Title/Abstract] OR "psoriatic arthritis"[Title/Abstract] OR "psoriasis arthropathica"[Title/Abstract] OR "psoriatic arthropathy"[Title/Abstract] OR "arthropathy psoriatic"[Title/Abstract] OR "psoriatic arthropathies"[Title/Abstract] OR "Polymyalgia Rheumatica"[MeSH Terms] OR "forestier certonciny syndrome"[Title/Abstract] OR "forestier certonciny syndrome"[Title/Abstract] | 11940318 |
| #4 | "preexisting"[Title/Abstract] OR "pre-existing"[Title/Abstract] OR "history"[Title/Abstract] OR "prior"[Title/Abstract] OR "previous"[Title/Abstract] OR "concomitant"[Title/Abstract] OR "concurrent"[Title/Abstract] OR "baseline"[Title/Abstract] | 3,362,360 |
| #5 | #1 AND #2 AND #3 AND #4 | 4,863 |
