# Supplementary material for: Immune checkpoint inhibitors in Cancer patients with rheumatologic preexisting autoimmune diseases: a systematic review and meta-analysis
Source: BMC Cancer. 2024 Apr 17;24:490. doi: 10.1186/s12885-024-12256-z (PMC11025164; doi:10.1186/s12885-024-12256-z)
Supplement: Supplementary file 2 — Supplementary Material 2 [file 12885_2024_12256_MOESM2_ESM.docx]

**Supplementary Table 2.** More characteristics of the studies included in the meta-analysis

| Author | Year | Country | Period | Study design | Type of cancer | Type of drug | Rheumatological disease subtype | Patients  (n) | Number of RA/other rheumatic diseases (n) | Age, median (range) years | Male  (%) | Median  follow-up day |
| --- | --- | --- | --- | --- | --- | --- | --- | --- | --- | --- | --- | --- |
| Bhatlapenumarthi | 2020 | North America | 2015-2019 | Retrospective | Mixed | Anti-PD-1/PD-L1 | Mixed | 24 | 12/12 | NA | NA | NA |
| Brown | 2021 | Multi-nation | 2015-2020 | Retrospective | Melanoma | Mixed | Mixed | 11 | 7/4 | NA | NA | 14 |
| Cortellini | 2019 | Europe | 2013-2018 | Retrospective | Mixed | Anti-PD-1/PD-L1 | Mixed | 10 | NA | NA | NA | 14.7 |
| Danlos | 2018 | Europe | 2014-2016 | Prospective | Mixed | Anti-PD-1/PD-L1 | Mixed | 7 | NA | NA | NA | 5.1 |
| Efuni | 2021 | North America | 2011-2018 | Retrospective | Mixed | Mixed | RA | 22 | NA | 67^a^ | 27 | NA |
| Gutzmer | 2017 | Europe | NA | Retrospective | Melanoma | Anti-PD-1/PD-L1 | Mixed | 9 | 1/8 | NA | NA | NA |
| Hoa | 2021 | North America | 2013-2019 | Retrospective | Mixed | Mixed | Mixed | 19 | 8/11 | NA | NA | 11 |
| Johnson | 2016 | Multi-nation | 2012-2015 | Retrospective | Melanoma | Anti-CTLA-4 | Mixed | 10 | 6/4 | NA | NA | NA |
| Kähler | 2017 | Europe | NA | Retrospective | Melanoma | Anti-CTLA-4 | Mixed | 14 | 6/8 | NA | NA | NA |
| Kaur | 2019 | North America | 2014-2016 | Retrospective | Mixed | Anti-PD-1/PD-L1 | Mixed | 5 | 4/1 | NA | NA | NA |
| Lee | 2016 | Australia | 2013-2014 | Retrospective | Melanoma | Anti-CTLA-4 | RA | 8 | NA | 72(53-84)^a^ | 38 | 13 |
| Leonardi | 2018 | North America | 2015-2017 | Retrospective | NSCLC | Anti-PD-1/PD-L1 | Mixed | 25 | 11/14 | NA | NA | 17.5 |
| Loriot | 2020 | Multi-nation | 2016-2018 | Prospective | Urological cancer | Anti-PD-1/PD-L1 | Mixed | 7 | 4/3 | NA | NA | NA |
| Lusa | 2022 | North America | 2014-2019 | Retrospective | Mixed | Mixed | Mixed | 45 | 22/23 | 67.9(±11.2)^b^ | 31 | NA |
| Machado | 2023 | North America | 2014-2021 | Retrospective | Mixed | Mixed | RA | 58 | NA | NA | NA | NA |
| Martinez Chanza | 2020 | Multi-nation | NA | Retrospective | Urological cancer | Mixed | Mixed | 35 | 12/23 | NA | NA | 15 |
| Menzies | 2016 | Multi-nation | 2012-2015 | Retrospective | Melanoma | Anti-PD-1/PD-L1 | Mixed | 27 | 13/14 | NA | NA | 4.7 |
| Mitchell | 2018 | Australia | 2014-2017 | Retrospective | Mixed | Anti-PD-1/PD-L1 | Mixed | 12 | 1/11 | NA | NA | 41 |
| Mooradian | 2019 | North America | 2014-2018 | Retrospective | Mixed | Mixed | Mixed | 6 | 2/4 | 67(42-81)^b^ | NA | 27 |
| Panhaleux | 2020 | Europe | 2019-2021 | Retrospective | Mixed | Anti-PD-1/PD-L1 | SSc | 17 | NA | 60(34-82)^a^ | 41 | 12 |
| Richter | 2017 | North America | 2011-2016 | Retrospective | Mixed | Anti-PD-1/PD-L1 | Mixed | 16 | 5/11 | 68.3^a^ | 19 | NA |
| Tison | 2019 | Europe | 2017-2018 | Retrospective | Mixed | Mixed | Mixed | 39 | 20/19 | NA | NA | 8 |
| Van der Kooij | 2021 | Europe | 2013-2019 | Prospective | Melanoma | Mixed | Mixed | 227 | NA | NA | NA | 18 |

Abbreviations: A, Median; b, Mean; NSCLC, non-small cell lung cancer; RA, rheumatoid arthritis; SSc, Systemic sclerosis; NA, not available.
