# Supplementary material for: Immune checkpoint inhibitors in Cancer patients with rheumatologic preexisting autoimmune diseases: a systematic review and meta-analysis
Source: BMC Cancer. 2024 Apr 17;24:490. doi: 10.1186/s12885-024-12256-z (PMC11025164; doi:10.1186/s12885-024-12256-z)
Supplement: Supplementary file 3 — Supplementary Material 3 [file 12885_2024_12256_MOESM3_ESM.docx]

**Supplementary Table 3.** Quality assessment of cohort studies by Newcastle–Ottawa Scale (NOS)

| Author | Selection | Comparability | Outcome | Toal |
| --- | --- | --- | --- | --- |
| Cortellini | 4 | 2 | 3 | 9 |
| Danlos | 4 | 0 | 3 | 7 |
| Loriot | 4 | 0 | 2 | 6 |
| Van der Kooij | 4 | 0 | 2 | 6 |
