# Supplementary material for: Immune checkpoint inhibitors in Cancer patients with rheumatologic preexisting autoimmune diseases: a systematic review and meta-analysis
Source: BMC Cancer. 2024 Apr 17;24:490. doi: 10.1186/s12885-024-12256-z (PMC11025164; doi:10.1186/s12885-024-12256-z)
Supplement: Supplementary file 4 — Supplementary Material 4 [file 12885_2024_12256_MOESM4_ESM.docx]

**Supplementary Table 4.** Quality assessment of case series by Joanna Briggs Institute (JBI)

| Author | Q1 | Q2 | Q3 | Q4 | Q5 | Q6 | Q7 | Q8 | Q9 | Q10 |
| --- | --- | --- | --- | --- | --- | --- | --- | --- | --- | --- |
| Bhatlapenumarthi | Yes | Yes | Yes | Yes | No | Yes | Yes | No | Yes | No |
| Brown | Yes | Yes | Yes | Yes | Yes | Yes | Yes | Yes | Yes | Yes |
| Efuni | Yes | Yes | Yes | Yes | No | Yes | Yes | No | Yes | Yes |
| Gutzmer | Yes | Yes | Yes | Yes | Yes | Yes | Yes | Yes | Yes | Yes |
| Hoa | Yes | Yes | Yes | Yes | Yes | Yes | Yes | Yes | Yes | No |
| Johnson | Yes | Yes | Yes | Yes | Yes | Yes | Yes | Yes | Yes | No |
| Kähler | Yes | Yes | Yes | Yes | Yes | Yes | Yes | Yes | Yes | No |
| Kaur | Yes | Yes | Yes | Yes | No | Yes | Yes | No | Yes | No |
| Lee | Yes | Yes | Yes | Yes | No | Yes | Yes | Yes | Yes | No |
| Leonardi | Yes | Yes | Yes | Yes | Yes | Yes | Yes | Yes | Yes | Yes |
| Lusa | Yes | Yes | Yes | Yes | No | Yes | Yes | Yes | Yes | Yes |
| Machado | Yes | Yes | Yes | Yes | No | Yes | Yes | No | Yes | No |
| Martinez Chanza | Yes | Yes | Yes | Yes | Yes | Yes | Yes | Yes | Yes | Yes |
| Menzies | Yes | Yes | Yes | Yes | Yes | Yes | Yes | Yes | Yes | Yes |
| Mitchell | Yes | Yes | Yes | Yes | Yes | Yes | Yes | Yes | Yes | Yes |
| Mooradian | Yes | Yes | Yes | Yes | No | Yes | Yes | No | Yes | No |
| Panhaleux | Yes | Yes | Yes | Yes | No | Yes | Yes | Yes | Yes | Yes |
| Richter | Yes | Yes | Yes | Yes | No | Yes | Yes | No | Yes | No |
| Tison | Yes | Yes | Yes | Yes | Yes | Yes | Yes | Yes | Yes | Yes |

Abbreviations: Q1, Were there clear criteria for inclusion in the case series? Q2, Was the condition measured in a standard, reliable way for all participants included in the case series? Q3, Were valid methods used for identification of the condition for all participants included in the case series? Q4, Did the case series have consecutive inclusion of participants? Q5, Did the case series have complete inclusion of participants? Q6, Was there clear reporting of the demographics of the participants in the study? Q7, Was there clear reporting of clinical information of the participants? Q8, Were the outcomes or follow up results of cases clearly reported? Q9, Was there clear reporting of the presenting site(s)/clinic(s) demographic information? Q10, Was statistical analysis appropriate?
