# Supplementary material for: Immune checkpoint inhibitors in Cancer patients with rheumatologic preexisting autoimmune diseases: a systematic review and meta-analysis
Source: BMC Cancer. 2024 Apr 17;24:490. doi: 10.1186/s12885-024-12256-z (PMC11025164; doi:10.1186/s12885-024-12256-z)
Supplement: Supplementary file 5 — Supplementary Material 5 [file 12885_2024_12256_MOESM5_ESM.docx]

| Flares | Coef | t | P | 95%CI |
| --- | --- | --- | --- | --- |
| Country (North America or Multi or Europe or Australia) | 0.122 | 2.29 | 0.035 | 0.009-0.234 |
| Type of drug (anti-PD-1/PD-L1 or anti-CTLA-4) | -0.858 | -0.81 | 0.429 | -0.309-0.137 |
| Sample size (over 20 or not) | 0.0323 | 0.29 | 0.774 | -0.201-0.266 |
| Study design (cohort or case series) | 0.316 | 1.41 | 0.175 | -0.155-0.788 |

**Supplementary Table 5.** Meta-regression for the outcome of flares
