# Supplementary figures and images for: Immune checkpoint inhibitors in Cancer patients with rheumatologic preexisting autoimmune diseases: a systematic review and meta-analysis
Source: BMC Cancer. 2024 Apr 17;24:490. doi: 10.1186/s12885-024-12256-z (PMC11025164; doi:10.1186/s12885-024-12256-z)

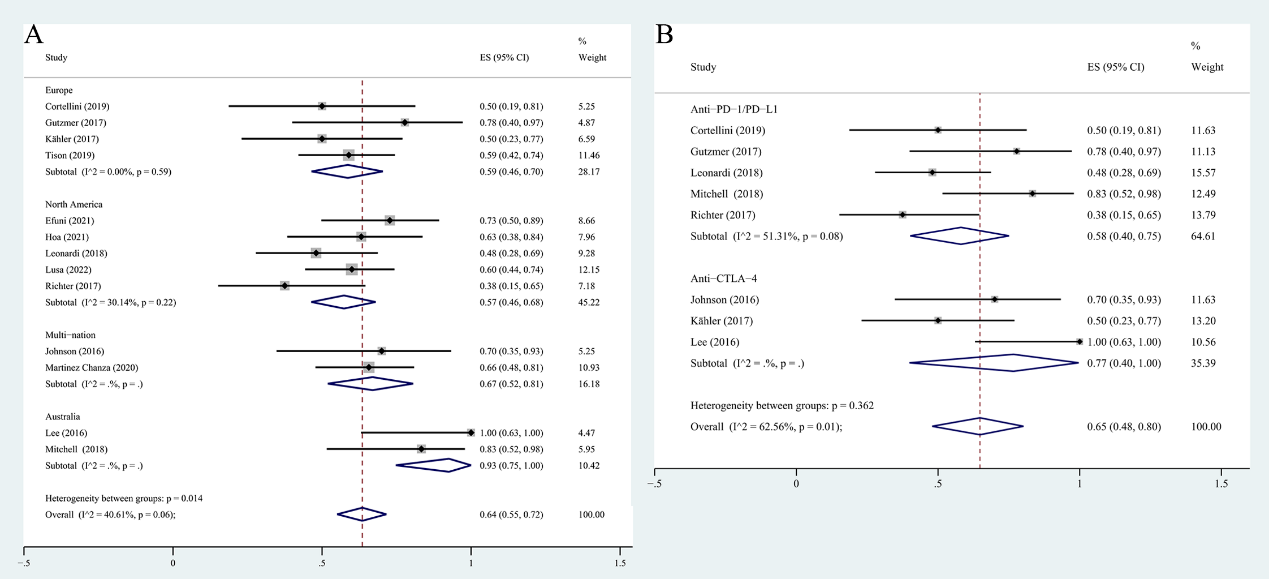


**Supplementary Fig. 1.** Subgroup analysis of any-grade TirAEs, stratified by: (A) region. (B) type of ICI

Supplement: Supplementary file 6 — Supplementary Material 6 [file 12885_2024_12256_MOESM6_ESM.docx]

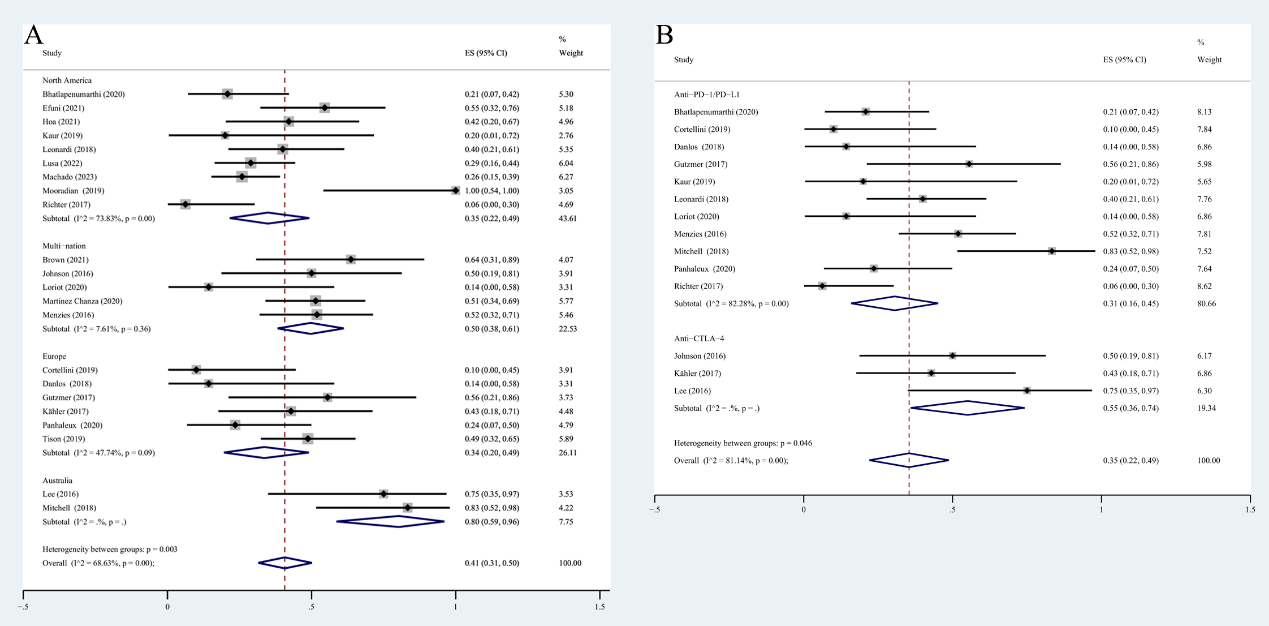


**Supplementary Fig. 3.** Subgroup analysis of any-grade flares, stratified by: (A) region. (B) type of ICI

Supplement: Supplementary file 8 — Supplementary Material 8 [file 12885_2024_12256_MOESM8_ESM.docx]

**
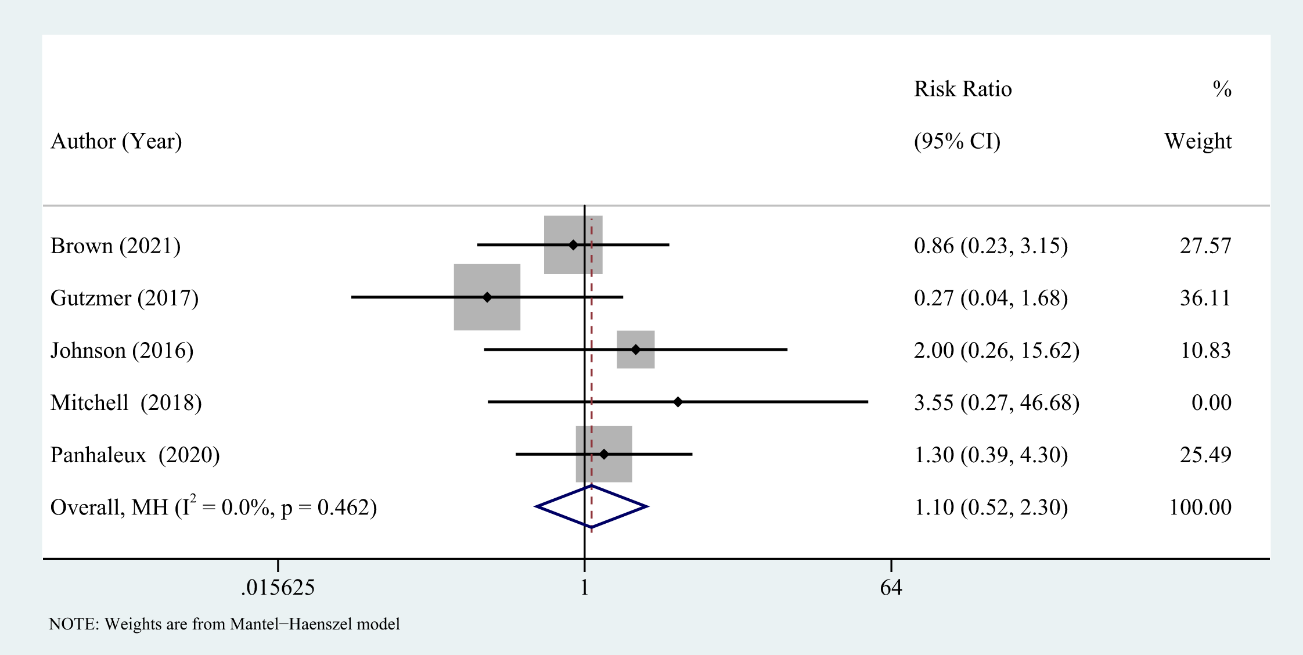
**

**Supplementary Fig. 5.** The assciation between flares and ORR

Supplement: Supplementary file 10 — Supplementary Material 10 [file 12885_2024_12256_MOESM10_ESM.docx]

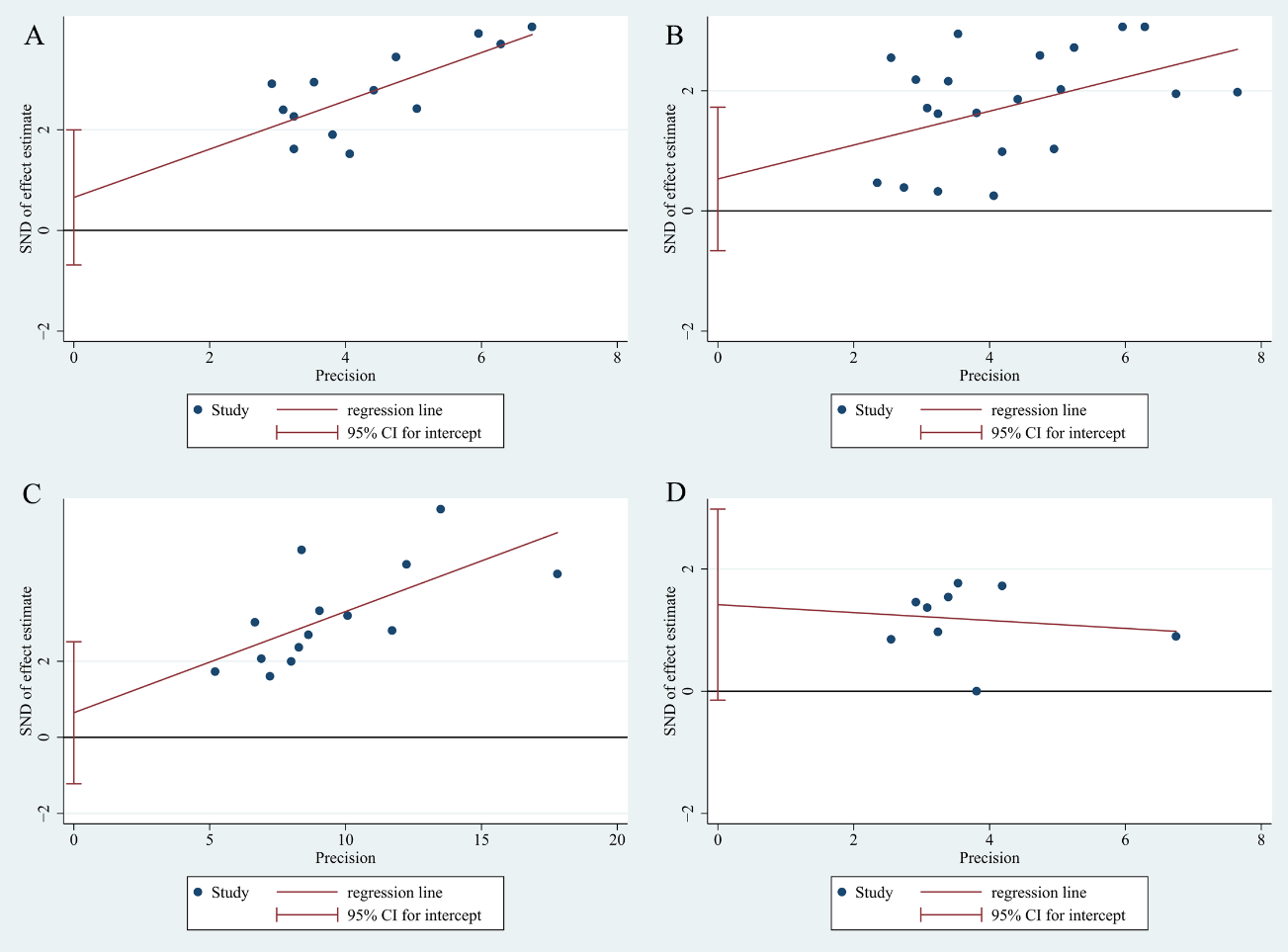
 **Supplementary Fig. 6.** Egger’s test for included studies. (A) TirAEs. (B) flares. (C) new onset irAEs. (D) ORR

Supplement: Supplementary file 11 — Supplementary Material 11 [file 12885_2024_12256_MOESM11_ESM.docx]

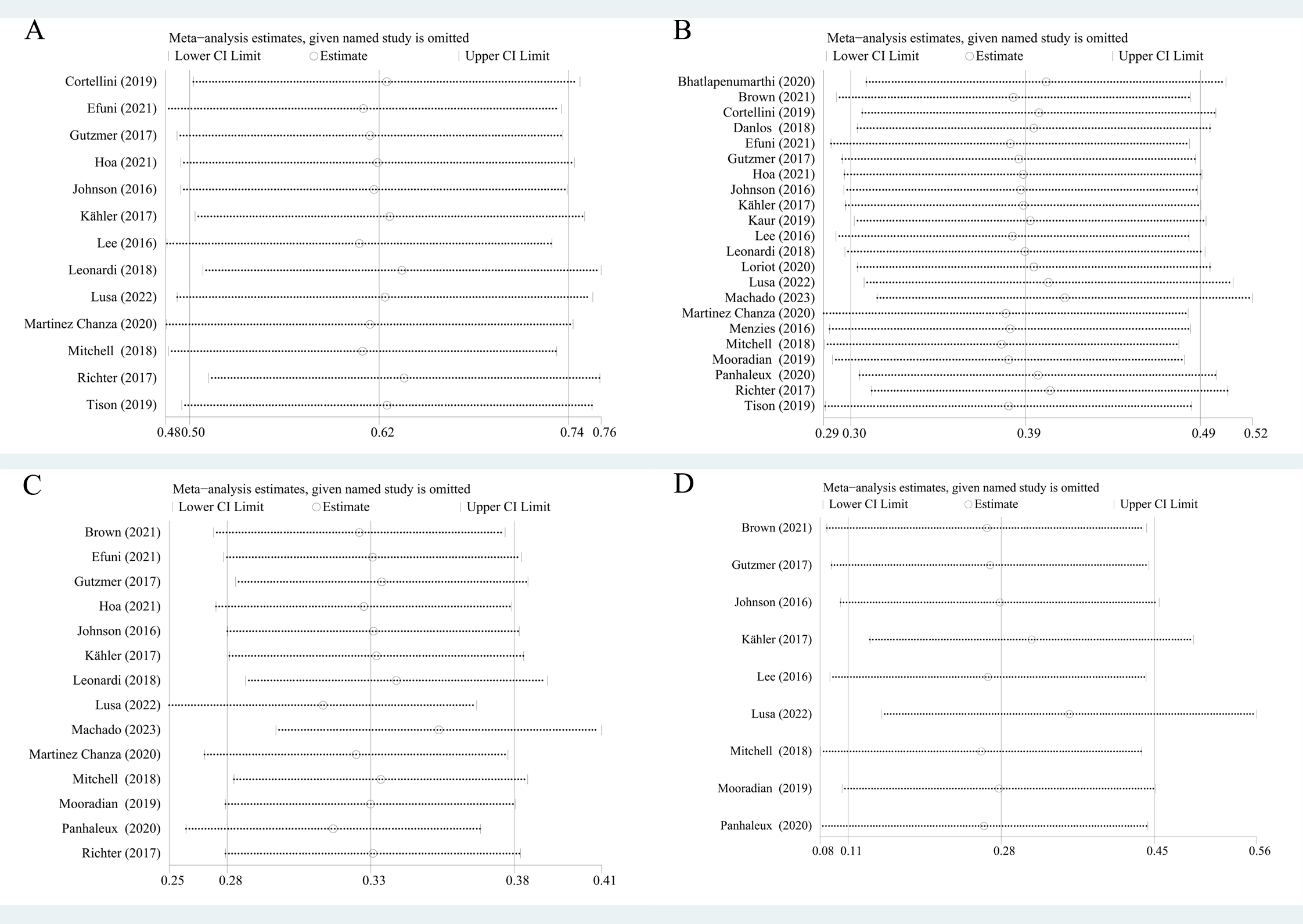


**Supplementary Fig. 7.** The results of sensitivity analyses. (A) TirAEs. (B) flares. (C) new onset irAEs. (D) ORR

Supplement: Supplementary file 12 — Supplementary Material 12 [file 12885_2024_12256_MOESM12_ESM.docx]
