# Supplementary material for: Immune checkpoint inhibitors in Cancer patients with rheumatologic preexisting autoimmune diseases: a systematic review and meta-analysis
Source: BMC Cancer. 2024 Apr 17;24:490. doi: 10.1186/s12885-024-12256-z (PMC11025164; doi:10.1186/s12885-024-12256-z)
Supplement: Supplementary file 7 — Supplementary Material 7 [file 12885_2024_12256_MOESM7_ESM.docx]

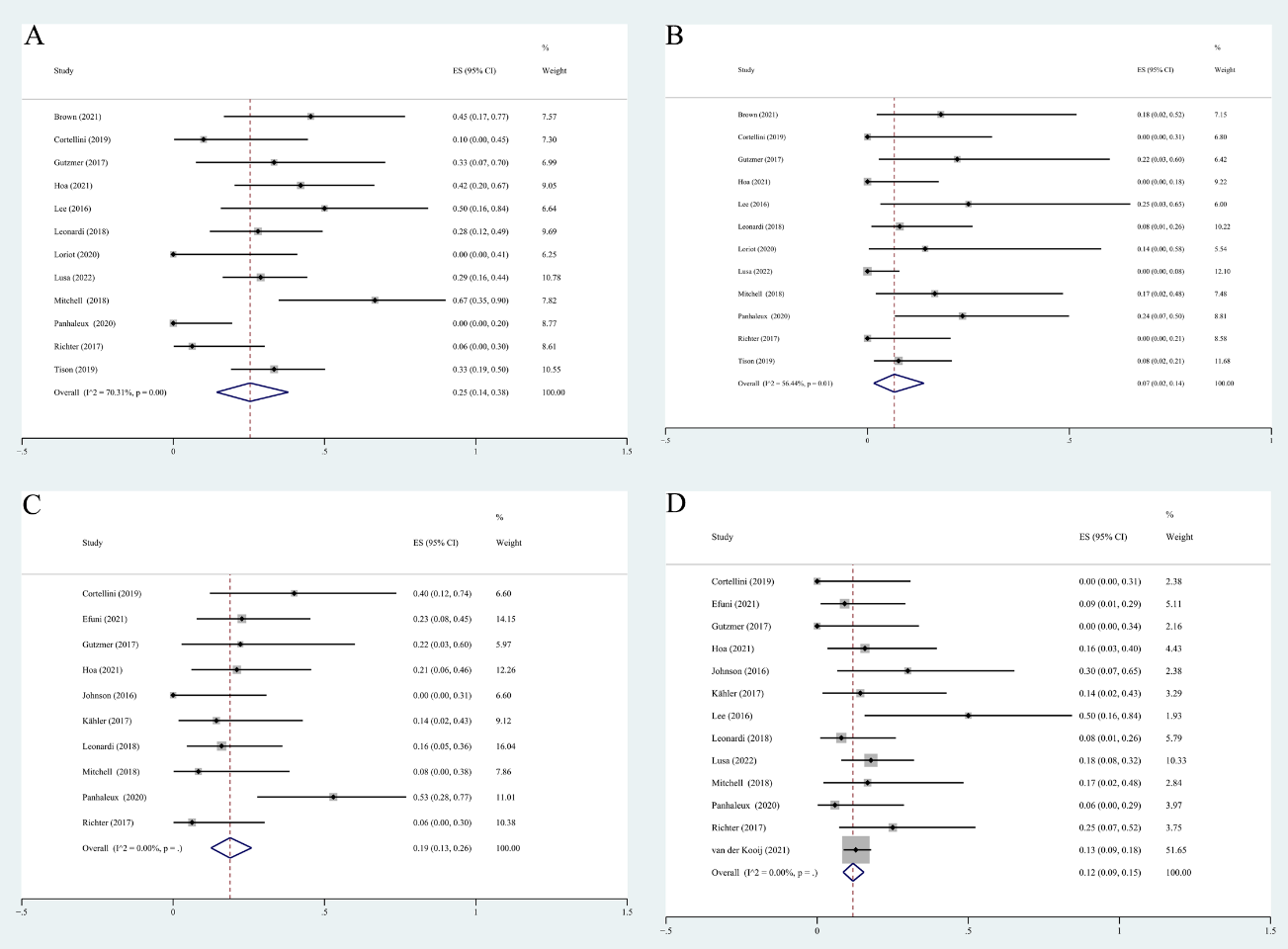


**Supplementary Fig. 2.** (A) The pooled incidence rates of flares grade 1-2. (B) The pooled incidence rates of flares grade 3-4. (C) The pooled incidence rates of new onset irAEs grade 1-2. (D) The pooled incidence rates of new onset irAEs grade 3-4
