# Supplementary material for: Immune checkpoint inhibitors in Cancer patients with rheumatologic preexisting autoimmune diseases: a systematic review and meta-analysis
Source: BMC Cancer. 2024 Apr 17;24:490. doi: 10.1186/s12885-024-12256-z (PMC11025164; doi:10.1186/s12885-024-12256-z)
Supplement: Supplementary file 9 — Supplementary Material 9 [file 12885_2024_12256_MOESM9_ESM.docx]

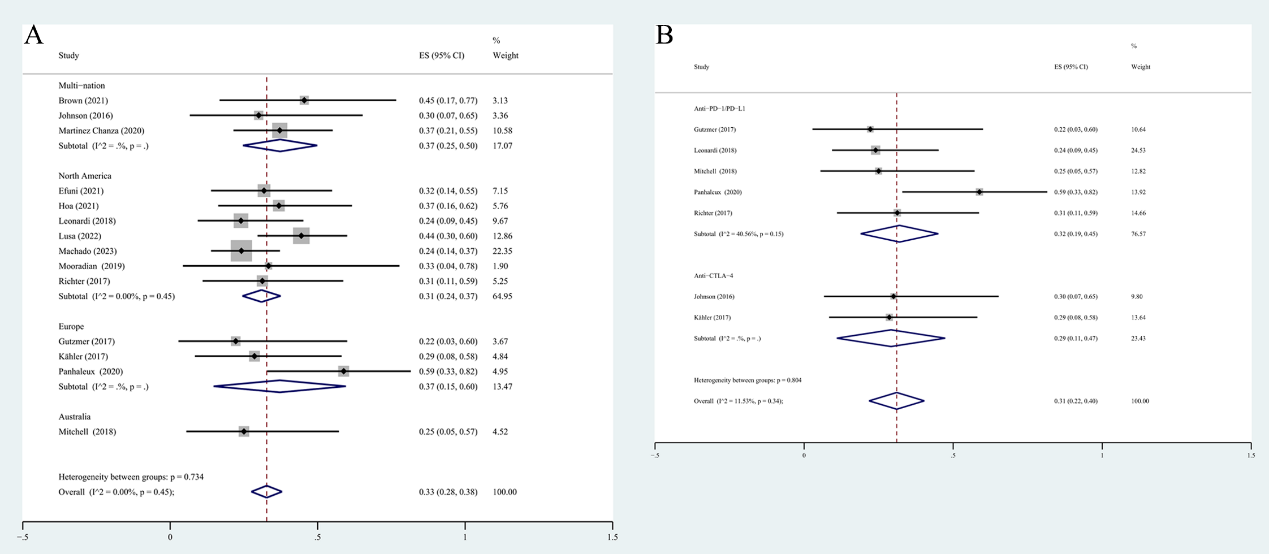


**Supplementary Fig. 4.** Subgroup analysis of any-grade new onset irAEs, stratified by: (A) region. (B) type of ICI
